# *N*-Salicyl-amino acid derivatives with antiparasitic activity from *Pseudomonas* sp. UIAU-6B

**DOI:** 10.3762/bjoc.21.103

**Published:** 2025-07-04

**Authors:** Joy E Rajakulendran, Emmanuel Tope Oluwabusola, Michela Cerone, Terry K Smith, Olusoji O Adebisi, Adefolalu Adedotun, Gagan Preet, Sylvia Soldatou, Hai Deng, Rainer Ebel, Marcel Jaspars

**Affiliations:** 1 Department of Chemistry, Faculty of Science, Eastern University, Vantharumoolai, Chenkalady, Sri Lanka; 2 Marine Biodiscovery Centre, Department of Chemistry, University of Aberdeen, AB24 3UE, Scotland, UK; 3 Schools of Biology & Chemistry, BSRC, The University of St. Andrews, Fife, KY16 9ST, UK; 4 Department of Microbiology, Faculty of Life Sciences, University of Ilorin, Kwara State, Ilorin, Nigeria; 5 Department of Biochemistry, Federal University of Lafia, Nigeria

**Keywords:** antiparasitic activity, biosynthesis pathway, phenolic siderophores, *Pseudomonas* species, pseudomonine

## Abstract

Pseudomonads strains represent a promising source of bioactive compounds with potential pharmaceutical applications. The necessity to find new drugs is underscored by the increased concern over antimicrobial resistance in the human system. In this study, we isolated two previously undescribed *N*-salicyl-amino acids as natural products (**1** and **2**) and other two new derivatives (**3** and **4**) from the organic extract of a culture broth in a modified starch–glucose–glycerol (SGG) medium of *Pseudomonas* sp. UIAU-6B. The structure of the new natural products, pseudomonins D–G (**1**–**4**) isolated alongside other three known compounds, pseudomonine (**5**), pseudomonin B (**6**) and salicylic acid (**7**), were elucidated based on high-resolution mass spectrometry, 1D and 2D NMR analyses. The absolute configurations of the threonine residue in compounds **1** and **2** were determined by Marfey’s analysis. Compound **4** displayed a very weak pan-trypanocidal activity against *Trypanosoma brucei, Trypanosoma cruzi and Leishmania major* with EC_50_ values of 101–137 μM, while compounds **2** and **5** showed modest activity against *Leishmania major,* but none of the remaining compounds showed activity at the highest concentrations tested. The plausible biosynthetic hypotheses toward the compounds were also proposed.

## Introduction

Species of the genus *Pseudomonas* are huge producers of bioactive natural products with a vast array of structural and functional molecular diversity. This reflects in the bacteria’s genome which ranges in size from 4.6 to 7.1 megabases with 4237 to 6396 predicted genes [1]. Natural products play a major role in the live of these bacteria, especially in the acquisition of nutrients [2], establishment of virulence [3–6], and defence against predators and competitors [7–8]. One such class of secondary metabolites are the siderophores [9–11] which are structurally diverse low molecular weight secondary metabolites released by microorganisms into their surrounding extracellular environment to chelate iron and selectively import it into the microbial cells [12–13]. It has been suggested that the release of siderophores by soil-dwelling pseudomonads give them an ecological edge by creating an iron-deprived environment in the soil and thereby inhibiting the growth and sporulation of several pathogenic microorganisms [14].

In our effort to discover new bioactive secondary metabolites, our group previously cultured the *Pseudomonas* UIAU-6B strain in a closed system at 28 °C, and the methanolic extract led to the isolation of five phenolic siderophores with some of them exhibiting antimicrobial properties, including pseudomonins A–C and pseudomobactins A and B [15]. In this study we report the isolation and structural characterization of two previously undescribed as natural products (**1** and **2**) and two new compounds (**3** and **4**) isolated alongside three known compounds, pseudomonine (**5**), pseudomonin B (**6**) and salicylic acid (**7**) [15] from the *Pseudomonas* sp. UIAU-6B strain by altering the fermentation conditions using an open system shaker at room temperature [16–18].

## Results and Discussion

**Figure 1:**
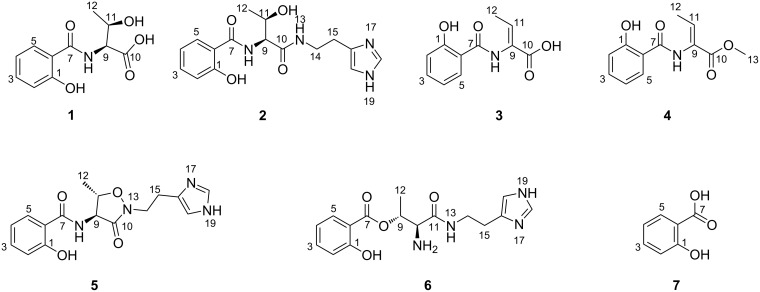
Structures of the pseudomonins D–G (**1**–**4**), pseudomonine (**5**), pseudomonin B (**6**) and salicylic acid (**7**).

The bacterial strain *Pseudomonas* sp. UIAU-6B was investigated by varying the temperature of the culture to elicit production of secondary metabolites after our previous work on the strain [15]. A small-scale culture of the strain (50 mL of SGG medium) shaken at 160 rpm in an open system shaker at room temperature yielded 0.008 g extract. The chemical dereplication of the crude extract by high-resolution electrospray ionisation mass spectrometry (HRMS) analysis and online natural product databases (Reaxys [19], AntiBase 2017 [20] and Natural Product Atlas [21]) revealed the presence of unknown molecular ions in addition to the previously isolated pseudomonine [15]. This resulted in the decision to upscale the bacterial strain at room temperature in an open orbital shaker. The large crude extract (7.5 g) was subjected to Kupchan solvent partitioning followed by medium pressure liquid chromatography (MPLC) and subsequently purification by a reversed-phase high pressure liquid chromatography (HPLC) system which led to the isolation of four compounds, trivially named pseudomonins D–G (**1–4**) and three known compounds **5**–**7** (Figure 1).

**Figure 2:**
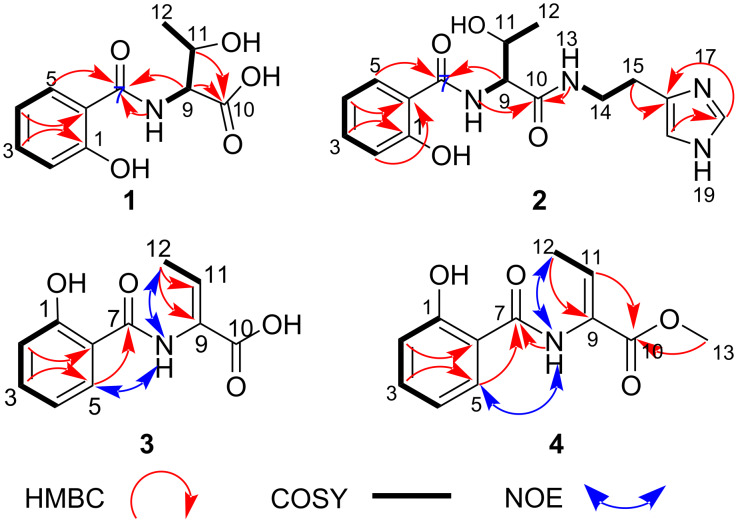
Key HMBC, ^1^H-^1^H COSY and NOE correlations.

Compound **1** was isolated as a yellow oil. The molecular formula C_11_H_13_NO_5_ (Δ: −0.7 ppm, 6 degrees of unsaturation, calcd. for C_11_H_12_NO_5_, 238.0721) of **1** was established from negative mode high-resolution electrospray ionisation (HRESI) mass spectrum data which showed a deprotonated molecular ion, [M − H]^−1^ at *m*/*z* 238.0723. The complete analysis of 1D and 2D NMR spectra (see Table 1 and Supporting Information File 1, Figures S1–S4) of **1** measured in deuterated DMSO revealed well-defined proton signals identified as four aromatic sp^2^ methines, two aliphatic sp^3^ methines, a methyl group and four quaternary carbon signals at δ_C_ 157.7 (C-1), δ_C_117.8 (C-6), δ_C_ 167.2 (C-7), and δ_C_ 172.9 (C-10). The presence of a 1,2-disubstituted benzene ring system was identified by the ^1^H NMR spectrum showing downfield methine protons at δ_H_ 7.88 (dd, *J* = 8.0, 1.5 Hz, H-5), 7.37 (td, *J* = 8.0, 1.5 Hz, H-3) 7.01 (d, *J* = 8.0 Hz, H-2) and 6.92 (t, *J* = 8.0, H-4) that were consistent with a salicylic acid unit. This was supported by a ^1^H-^1^H COSY spectrum showing correlations from H-2/H-3, H-3/H-4 and H-4/H-5 (see Figure 2). The second spin system corresponding to protons including exchangeable NH signal H-8 (δ_H_ 8.82, d, *J* = 6.9 Hz), H-9 (δ_H_ 4.41, dd, *J* = 6.9, 6.4 Hz), oxygenated H-11 (δ_H_ 4.24, qd, *J* = 6.9, 6.4 Hz) and H-12 (δ_H_ 1.10, d, *J* = 6.9 Hz) revealed the presence of a threonine moiety in **1**. Strong HMBC correlations from H-5, H-8 and H-9 to the carbonyl carbon C-7 (δ_C_ 167.2) confirmed an amide bond by which the salicylic acid and threonine residues were linked. The deshielded carbon signal at δ_C_ 172.9 was assigned to C-10 showing the presence of the carboxyl functionality of the threonine moiety. Based on this conclusion, the structure of **1** was elucidated and named pseudomonin D.

**Table 1:** ^1^H and ^13^C NMR data of pseudomonins D–G (**1**–**4**).

	Compound **1**		Compound **2**		Compound **3**		Compound **4**	
Position	δ_C_, type	δ_H_, multiplicity (*J* in Hz)	δ_C_, type	δ_H_, multiplicity (*J* in Hz)	δ_C_, type	δ_H_, multiplcity (*J* in Hz)	δ_C_, type	δ_H_, multiplicity (*J* in Hz)

1	157.7, C		157.8, C		159.5, C		159.3, C	
2	117.3, CH	7.01, d (8.0)	117.3, CH	6.95, m	117.6, CH	6.95, d (7.9)	117.6, CH	6.96, m
3	134.1, CH	7.37, td (8.0, 1.5)	133.7, CH	7.38, td (8.0, 1.2)	134.5, CH	7.44, t (7.9)	134.4, CH	7.45, t (7.9)
4	120.1, CH	6.92, t (8.0)	119.6, CH	6.92, m	119.3, CH	6.94, t (7.9)	119.3, CH	6.94, m
5	130.6, CH	7.88 dd (8.0, 1.5)	130.3, CH	7.93, dd (8.0, 1.2)	129.0, CH	7.97, d (7.9)	129.0, CH	7.96, d (7.9)
6	117.8, C		117.8, C		115.8, C		115.6, C	
7	167.2, C		166.9, C		166.9, C		167.7, C	
8		NH, 8.82, d (6.9)		NH, 8.74, d (6.9)		NH, 9.82, s	–	NH, 9.9, s
9	58.5, CH	4.41, dd (6.9, 6.4)	59.5, CH	4.28, dd (6.9, 6.4)	128.1, C		127.5, C	
10	172.9, C		170.7, C		173.5, C		164.7, C	
11	66.8, CH	4.24, qd (6.9, 6.4)	66.7, CH	4.07, (6.9, 6.4)	133.9, CH	6.73, q (7.1)	134.4, CH	6.73, q (6.9)
12	21.0, CH_3_	1.10, d (6.9)	20.6, CH_3_	1.06, d (6.9)	14.0, CH_3_	1.74, d (7.1)	13.8, CH_3_	1.76, d (6.9)
13				NH, 8.1, t (6.7)			52.3, CH_3_	3.68, s
14			37.9, CH_2_	3.38, m				
15			24.5, CH_2_	2.8, t (6.7)				
16			131.4, C					
18			133.8, CH	8.97, s				
20			116.5, CH	7.41, s				

Compound **2** was obtained as a reddish oil, and the molecular formula deduced as C_16_H_20_N_4_O_4_ (Δ: +0.4 ppm, calcd. for C_16_H_21_N_4_O_4_, 333.1557) with 9 degrees of unsaturation based on positive mode HRESIMS data which indicated a protonated molecular ion, [M + H]^+^ at *m*/*z* 333.1556 (see Supporting Information File 1, Figure S6). Analysis of 1D and 2D NMR spectra (see Table 1 and Supporting Information File 1, Figures S7–S9) of **2** revealed resonance signals characteristic of salicylic and threonine units in **1**. Besides, some other resonance signals were observed, including one exchangeable NH signal at δ_H_ 8.10 (t, *J* = 6.7 Hz, H-13), two sp^3^ methylenes signal at δ_H_ 3.38 (H_2_-14, m) and δ_H_ 2.80 (t, *J* = 6.7 Hz, H_2_-15) , two aromatic sp^2^ methine signal at δ_H_ 8.97 (s, H-18), δ_H_ 7.41 (s, H-20) and a quaternary carbon (C-16) assigned at δ_C_ 131.4 that reminiscences a histamine subunit absent in compound **1**. This observation was supported by the ^1^H-^1^H COSY spectrum that displayed a cross-peak coupling of H_2_-14 to both carboxamide protons H-13 and H_2_-15. Strong HMBC correlations observed from H-18 to C-16, C-20 (δ_H_ 116.5) and from H-20 to C-18 (δ_H_ 133.8) and H-15 to C-16 (see Figure 2) confirmed the presence of a monosubstituted imidazole subunit of histamine linked to the methylene groups at C-16 as evidenced by a two-bond correlation from H-15 to C-16. Compound **2**, trivially named pseudomonin E, was similar to the previously reported compound, pseudomonine except for the opening of the oxazolidinone ring of its threonine unit [22]. Compounds **1** and **2** have been synthetically produced in the reconstruction of the enzymatic pathway to explain the biogenesis of this heterocyclisation leading to the pseudomonine biosynthesis [23].

The absolute configuration of the threonine residue in compounds **1** and **2** was assigned as ʟ, based on the derivatization of **1** and **2** hydrolysates with ʟ-FDAA, a Marfey’s reagent, followed by chromatographic profiling by HPLC with a wavelength monitored at 340 nm of the derivatized amino acid residuals in the hydrolysate and threonine standards (see Supporting Information File 1, Figure S24).

Compound **3** was isolated as a yellow oil. Its molecular formula of C_11_H_11_NO_4_ (Δ: −0.3 ppm, calcd. for C_11_H_10_NO_4_, 220.0615) with 7 degrees of unsaturation was deduced based on negative mode HRESIMS data (see Supporting Information File 1, Figure S11) which indicated a deprotonated anion at *m/z* 220.0616. The ^1^H NMR spectrum of **3** displayed slightly similar signal patterns to that of **1** but differs due to dehydration of the threonine unit. This results in the absence of a sp^3^ proton of the α-carbon atom C-9 (δ_C_ 128.1) and a downfield chemical shift β-proton at δ_H_ 6.73 (*J*= 7.1 Hz, H-11) which was found to be vicinally coupled with the methyl protons, H_3_-12 (δ_H_ 1.74, d, *J* = 7.1 Hz). This was supported by the HMBC cross peak from H-11 to C-10 (δ_C_ 173.5), H_3_-12 to C-9 (δ_C_ 128.1) and C-11 (δ_C_ 133.9).

The relative stereochemistry of the double bond of the dehydrobutyrine moiety was established as *Z* based on a medium NOE correlation observed between the amide proton, H-8 (δ_H_ 9.82) and the methyl protons, H_3_-12 (see Figure 2 and Supporting Information File 1, Figure S16) confirming that both of these protons were present on the same side of the double bond. The structure of **3** was confirmed using mass spectrometry, 1D and 2D NMR data as a new compound, named pseudomonin F.

Compound **4** was isolated as a pink oil. The negative mode HRESIMS gave a prominent molecular ion at *m/z* 234.074 corresponding to C_12_H_13_NO_4_ (Δ: +1.2 ppm, calcd. for C_12_H_12_NO_4_, 234.0772) having 7 degrees of unsaturation. Detailed analysis of the ^1^H NMR data of **4** showed similar resonance signals observed in **3**, however, an additional intense sharp singlet at δ_H_ 3.68 (H_3_-13) with an integral of three was observed in **4** confirming a methoxy group replacement. This was evidenced in an upfield resonance signal in C-10 (δ_C_ 164.7) and a strong HMBC cross peak correlation from H_3_-13 to C-10 in relation to **3**. The dehydrobutyrine moiety of **4** displayed the same relative configuration to that of **3**, evidenced by a medium through space correlation between the amide proton, H-8 (δ_H_ 9.9) and the methyl, H_3_-12 (δ_H_ 1.76) in the NOESY spectrum (see Figure 2 and Supporting Information File 1, Figure S23). Based on HRESIMS, 1D and 2D NMR data, the structure of compound **4**, was confirmed as a new compound, named pseudomonin G, which displays an unusual, dehydrobutyrine moiety that has not been previously reported.

**Figure 3:**
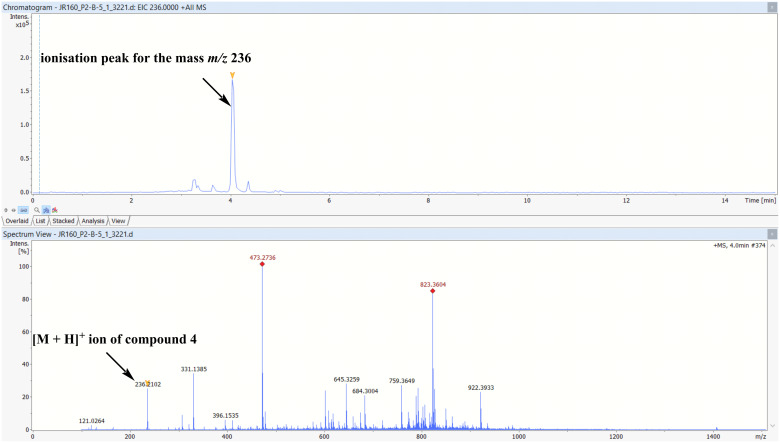
Extracted ion chromatogram and corresponding mass spectrum of compound **4** in the crude extract.

To confirm whether **4** was actually produced by the microbe and not an artefact formed due to spontaneous methylation by MeOH, an extracted ion chromatogram (Figure 3) for the mass, *m*/*z* 236, [M + H]^+^ was generated from LC–MS data of the crude extract of the large-scale bacterial culture. The chromatogram clearly revealed an ionization with a corresponding *m*/*z* of 236.2102 in the mass spectrum, thereby confirming the presence of **4** in the crude extract.

Compound **5** was isolated as a reddish oil and displayed a molecular ion in the positive mode HRESIMS at *m/z* 331.1403 from which its molecular formula, C_16_H_18_N_4_O_4_ (Δ: +0.7 ppm, 10 degrees of unsaturation, calcd. for C_16_H_19_N_4_O_4_, 331.1401) was established. The ^1^H NMR of **5** measured in DMSO-*d*_6_ displayed 13 proton signals which were consistent with that of the ^1^H NMR data of the previously isolated compound, pseudomonine and was further confirmed by the MS, 1D and 2D NMR data (see Supporting Information File 1, Figures S24–S28).

Compound **6** was isolated as a pinkish oil whose molecular formula was established as C_16_H_20_N_4_O_4_ based on the positive mode HRESIMS data which yielded a protonated molecular ion, [M + H]^+^ at *m/z* 333.1557 (Δ: 0 ppm, calcd. for C_16_H_21_N_4_O_4_, 333.1557) having 9 degrees of unsaturation. Comparison of MS, 1D and 2D NMR data (see Supporting Information File 1, Figures S29–S33) of **6** with our previously isolated compounds confirmed **6** as pseudomonin B.

Compound **7** was isolated as a pinkish crystalline solid which displayed a deprotonated anion, [M − H]^−^ in the negative mode HRESIMS at *m/z* 137.0213 from which the molecular formula of **7** was established as C_7_H_6_O_3_ (Δ: +0.1 ppm, 5 degrees of unsaturation, calcd. for C_7_H_5_O_3_, 137.0244). A comparison of MS, 1D and 2D NMR data (see Supporting Information File 1, Figures S34–S36) of **7** with literature data of salicylic acid was consistent.

**Figure 4:**
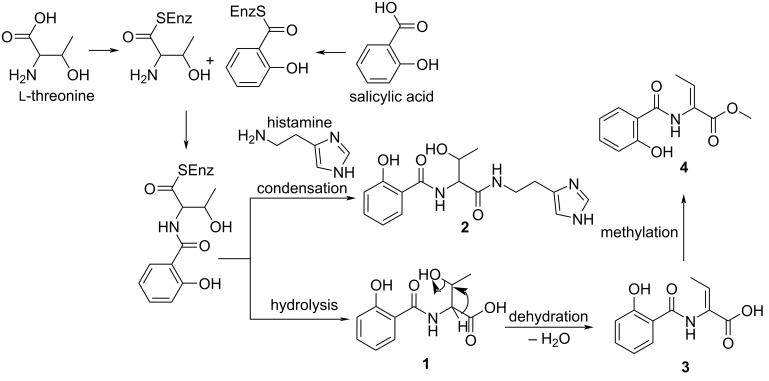
Proposed biosynthetic scheme for the formation of compounds (**1**–**4**).

Considering the structures of the previously undescribed phenolic siderophores (**1–4**), it is logical to assume that their tentative biosynthesis follows that of pseudomonine, a non-ribosomally synthesized isoxazolidone produced by *P. fluorescens.* The previous studies on the biosynthesis revealed seven genes, three non-ribosomal peptide synthases (Pms D, E, G) and four precursor biosynthetic enzymes (Pms A, B, C, F) [23–25]. The biosynthetic route of compound **1** leads to the amide-bond formation step between the PmsG-tethered threonine and salicylic acyl group leading to intramolecular dehydration. The decarboxylated product of histidine, histamine serves as a nucleophilic substrate to release the corresponding uncyclised condensation product, compound **2**. Compound **1** is proposed to further undergo a dehydration reaction by the elimination of the α-proton and β-hydroxy group of the threonine residue to form compound **3**. Compound **4** is a product of methylation of **3** carboxyl functionality (see Figure 4). These compounds are possibly siderophores because the isolates share common structural scaffold and are analogues of previously confirmed siderophores, including pseudomonine and salicylic acid [26–28] which were also isolated from the same culture as well. The experimental work to confirm these types of compounds as siderophores was previously described. Even, salicylic acid, a precursor in the biosynthesis of salicylate-derived siderophores confirmed the metallophore tendencies of the isolated compounds produced in iron-deficient environments [29–31].

### Biological evaluation

Compounds **1–7** were evaluated for their antiparasitic activity against *Trypanosoma brucei, Trypanosoma cruzi* and *Leishmania major*. Only compound **4** displayed a very weak pan-trypanocidal activity with EC_50_ values of 137, 127 and 101 μM against *T. brucei, T. cruzi* and *L. major,* respectively (see Table S1, Supporting Information File 1). Compounds **2** and **5** also show modest activity against *L. major.* All the remaining compounds (**1**–**3** and **5**–**7**) tested in this assay showed no activity at the highest concentration very likely due to the free carboxylic acid moiety present in some compounds (**1, 3** and **7**), prevent uptake into the parasites. This could also explain the observed differences in the EC_50_ values between the same structures **3** and **4**, except **4** is the methyl ester of **3**.

## Conclusion

In this study, we isolated four compounds, two previously undescribed as natural products (**1** and **2**) and two new compounds (**3** and **4**) from *Pseudomonas* sp. UIAU-6B cultured in SGG liquid medium at room temperature. The four compounds were characterised, and the absolute configurations of the threonine residues were determined as ʟ by Marfey’s analysis. A biosynthetic scheme was proposed for the formation of the isolated natural products. Compound **4** displayed a very weak pan-trypanocidal activity, this could be due to the “Michael acceptor” potential of the conjugated double bond. Additionally, for *T. brucei* this could be due to the salicylhydroxamic acid like structure, where salicylhydroxamic acid is known to inhibit the alternative oxidase found in bloodstream *T. brucei* [32]. Only compounds **2** and **5** showed modest anti-*L. major* activity, while all other compounds displayed no activity. This study shows the *Pseudomonas* strain as a reservoir of untapped secondary metabolites and as a potential source for isolating unique bioactive molecules.

## Experimental

### General experimental procedures

NMR spectra were recorded on a Bruker AVANCE III spectrometer with a Prodigy liquid nitrogen cryoprobe at 600 MHz. Samples were analysed in both positive and negative ion-modes electrospray ionisation using an MS system (Bruker MAXIS II equipped with a Quadrupole-Time-of-Flight mass analyzer) coupled to an HPLC (Agilent 1290 Infinity equipped with a diode array detector) on a Phenomenex analytical C18 column (2.5 µm, 100 Å, 4.6 × 150 mm). Samples were eluted with a starting mobile phase of 5% acetonitrile (MeCN):95% H_2_O (0.1% formic acid), followed by a gradient of up to 100% MeCN for 15 min at a flow rate of 1 mL/min. MS parameters were as follows: mass range *m/z* 100–2000, capillary voltage 4.5 kV, nebulizer gas 5.0 bar, dry gas 12.0 L/min, and dry temperature of 220 °C. MS/MS experiments were conducted in auto MS/MS scan mode with no step collision. The raw data file was analysed using Compact Data Analyst (Bruker software) to provide accurate and high-resolution mass per charge of molecular ions in the sample and generate molecular formulae using a Bruker Smart Formula manually.

### Microbial isolation and identification

The *Pseudomonas* sp. UIAU-6B strain was isolated from a sediment sample collected off the heavy metal-contaminated Oyun River in Nigeria. The isolation and identification of the bacterial strain UIAU-6B has been previously reported [15].

### Small-scale culture

Pure colonies of the *Pseudomonas* sp. UIAU-6B were inoculated into 100 mL of SGG (10 g of glucose, 10 g of glycerol, 2.5 g of corn steep solids, 5 g of peptone, 10 g of soluble starch, 2 g of yeast extract, 3 g of CaCO_3_ and 1 g of NaCl) medium at pH 7.0 in 250 mL Erlenmeyer flasks and cultured on an open shaker (160 rpm) at room temperature for 10 days. The corresponding blank media were also maintained under the same culture conditions as negative controls. At the end of incubation autoclaved Diaion HP‐20 resin (3 g/50 mL) was added to the small-scale cultures and the respective blank media and incubated (150 rpm) for another 24 h, filtered under a vacuum and subsequently extracted with 100% methanol. The extract was concentrated under reduced pressure and later dried completely in a nitrogen drier to yield 0.008 g extract.

### Large-scale fermentation

A pure colony of the strain *Pseudomonas* sp. UIAU-6B was inoculated into 100 mL SGG liquid medium in a 250 mL Erlenmeyer flask and shaken (150 rpm) for 3 days at room temperature. After the liquid culture was observed to have an increased bacterial growth, 2 mL of the seed culture was measured from the latter and inoculated into ten 1 L Erlenmeyer flasks containing 500 mL of autoclaved SGG medium and incubated at room temperature in an open orbital shaker (150 rpm) for 14 days. At the end of fermentation, 30 g of autoclaved Diaion HP‐20 resin was added to each flask and allowed to shake for 24 hours before the resin was filtered and subsequently extracted with methanol (500 mL × 3) followed by ethyl acetate (300 mL × 2). The combined organic extract was dried under reduced pressure, to yield 7.5 g of crude extract.

### Fractionation and isolation of compounds **1**–**4**

The crude extract (7.5 g) was subjected to fractionation by Kupchan partitioning [33]. The extract was dissolved in water (300 mL) and dichloromethane (300 mL × 3) and placed in a separating funnel for 5 min to allow for proper separation of the constituent mixture between the aqueous and organic phases. Further partitioning of the aqueous phase with *sec*-butanol (300 mL × 3) yielded FB fraction (2.3 g) and FW fraction (1.4 g), respectively. The dichloromethane (FD) fraction (3.5 g) was chromatographed using a medium performance liquid chromatography (MPLC) system equipped with a reversed-phase EcoFlex C18 cartridge (80 g, 50 µm) with variable UV and ELSD detectors to monitor the run at 210, 230, and 254 nm wavelengths. It was dissolved in 30–70% H_2_O/MeOH (15 mL), centrifuged for 5 min and gradually injected through the injection valve after the column washing and equilibration. The mobile phase gradient started with 30–70% H_2_O/MeOH for 35 min followed by an isocratic elution for 7 min on 15–85% H_2_O/MeOH, and a linear gradient elution to 100% MeOH for 30 min and subsequently maintained at 100% MeOH for further 20 minutes at the flow rate of 10 mL /min with a combined running time of 92 min. The MPLC run afforded the following subfractions: FD-1 (1.82 g), FD-2 (1.31 g), FD-3 (0.12 g), FD-4 (0.11 g) and FD-5 (0.10 g). FD-1 (1.82 g) was further purified using an HPLC system equipped with a preparative SunFire C18 reversed-phase column (10 µm, 10 × 250 mm) and eluted with 30% H_2_O/ 70% MeOH followed by a linear gradient elution scheme to 100% MeOH for 30 min at a flow rate of 2 mL/min affording **2** (2.32 mg, *t*_R_ 15.7 min) and **1** (4.13 mg, *t*_R_ 25.6 min). The FB fraction (2.3 g) was chromatographed using the same MPLC system. FB fraction (2.3 g) was dissolved in 60–40% H_2_O/MeOH (15 mL), centrifuged for 5 min and gradually injected through the injection valve after the column washing and equilibration.

The MPLC run started with the combination of isocratic and linear gradient solvent system of 60–40% H_2_O/MeOH for 38 min followed by an isocratic elution for 7 min on 20–80% H_2_O/MeOH. Then a linear gradient elution to 100% MeOH for 35 min and finally maintained at 100% MeOH for 20 min at the flow rate of 10 mL/min with a combined running time of 100 min. The MPLC run afforded the following subfractions: FB-1 (0.1 g), FB-2 (1.8 g) and FB-3 (0.1 g). Sub fraction FB-2 (1.8 g) was subjected to HPLC purification and eluted with 60% H_2_O/40% MeOH followed by a linear gradient elution scheme to 100% MeOH for 30 min at a flow rate of 2 mL/min to afford **3** (0.71 mg, *t*_R_ 20.5 min) and **4** (2.45 mg, *t*_R_ 28.6 min).

Pseudomonin D (**1**): Yellow oil; HRESIMS (*m/z*): [M − H]^−^ calcd. for C_11_H_12_NO_5_, 238.0721; found 238.0723(Δ: −0.7 ppm); NMR data, see Table 1 and Supporting Information File 1, Figures S1–S5 [23].

Pseudomonin E (**2**): Reddish oil; HRESIMS (*m/z*): [M + H]^+^ calcd. for C_16_H_21_N_4_O_4_, 333.1557; found, 333.1556 (Δ: +0.4 ppm); NMR data, see Table 1 and Supporting Information File 1, Figures S6–S10 [23].

Pseudomonin F (**3**): Yellow oil; HRESIMS (*m/z*): [M − H]^−^ calcd. for C_11_H_10_NO_4_, 220.0615; found, 220.0616 (Δ: −0.3 ppm); NMR data, see Table 1 and Supporting Information File 1, Figures S11–S17.

Pseudomonin G (**4**): Pink oil; HRESIMS (*m/z*): [M − H]^−^ calcd. for C_12_H_12_NO_4_, 234.0772; found, 234.074 (Δ: +1.2 ppm); NMR data, see Table 1 and Supporting Information File 1, Figures S18–S23.

Pseudomonine (**5**): Reddish oil; ; HRESIMS (*m/z*): [M + H]^+^ calcd. for C_16_H_19_N_4_O_4_, 331.1401; found, 331.1403; NMR data see Supporting Information File 1, Figures S24–S28 [22].

Pseudomonin B (**6**): Pinkish oil; ; HRESIMS (*m/z*): [M + H]^+^ calcd. for C_16_H_21_N_4_O_4_, 333.1557; found, 333.1557; NMR data see Supporting Information File 1, Figures S29–S33) [15].

Salicylic acid (**7**): Pinkish crystalline solid; HRESIMS (*m/z*): [M − H]^−^ calcd. for C_7_H_5_O_3_, 137.0244; found 137.0213; NMR data see Supporting Information File 1, Figures S34–S36) [15].

### In vitro cytotoxicity viability test for *T. brucei*

The bloodstream form of *T. brucei* strain 427 was grown in modified HMI-9 media as previously described [2]. The EC_50_ value of each compound (dissolved in DMSO) was determined using two-fold serial dilutions of the compound in media, carried out in 96-well plates in quadruplicate. Parasites were counted using a CASY TT Cell Counter. Alamar Blue viability assays were performed, whereby cells were incubated with the compound in serial dilution (specific details for each cell line are given below), after which the Alamar Blue cell viability reporter was added and the fluorescence recorded using an FLx 800 plate reader (BioTek) with excitation wavelength 535/540 nm and emission wavelength at 590/610 nm using Gen5 Reader Control 2.0 Software (BioTek). EC_50_ values were determined using a 4-parameter non-linear logistic regression equation using GraFit 5.0 (Erithacus Software) [3].

The bloodstream form of *T. brucei* stain Lister 427 was maintained in HMI-9 growth medium (IMDB medium supplemented with 10% (v/v) foetal bovine serum (FBS), 20% (v/v) Serum Plus, 0.05 mM bathocuproine sulfonate, 1.5 mM ʟ-cysteine, 1 mM hypoxanthine, 0.2 mM 2-mercaptoethanol, 1 mM sodium pyruvate and 0.16 mM thymidine). Cells were added to the compound (2-fold serially diluted in HMI-9 growth medium) to a density of 1 × 10^3^ cells/well and incubated at 37 °C with 5% CO_2_ for 72 hours, after which 20 μL Alamar Blue (0.125 mg/mL resazurin salt in PBS) was added to all wells and incubated for a further 6 hours before fluorescence recording.

## Supporting Information

File 1 Experimental information of the 1D and 2D NMR and LC–MS analyses (Figures S3–14), Marfey experiment (Figure S15) and the bioassay screen result (Figure S16).

## Data Availability

All data that supports the findings of this study is available in the published article and/or the supporting information of this article.
