# Supplementary material for: N-Salicyl-amino acid derivatives with antiparasitic activity from Pseudomonas sp. UIAU-6B
Source: Beilstein J Org Chem. 2025 Jul 4;21:1388–96. doi: 10.3762/bjoc.21.103 (PMC12230329; doi:10.3762/bjoc.21.103)
Supplement: File 1 — Experimental information of the 1D and 2D NMR and LC–MS analyses (Figures S3–14), Marfey experiment (Figure S15) and the bioassay screen result (Figure S16). [file Beilstein_J_Org_Chem-21-1388-s001.pdf]

BEILSTEIN JOURNAL OF ORGANIC CHEMISTRY

Supporting Information

for

N-Salicyl-amino acid derivatives with antiparasitic activity from *Pseudomonas* sp. UIAU-6B

Joy E. Rajakulendran, Emmanuel Tope Oluwabusola, Michela Cerone, Terry K. Smith, Olusoji O. Adebisi, Adefolalu Adedotun, Gagan Preet, Sylvia Soldatou, Hai Deng, Rainer Ebel and Marcel Jaspars

Beilstein J. Org. Chem. **2025,** *21,* 1388–1396. doi:10.3762/bjoc.21.103

Experimental information of the 1D and 2D NMR and LC–MS analyses (Figures S3–14), Marfey experiment (Figure S15) and the bioassay screen result (Figure S16)

License and Terms: This is a supporting information file under the terms of the Creative Commons Attribution License (https://creativecommons.org/licenses/by/4.0). Please note that the reuse, redistribution and reproduction in particular requires that the author(s) and source are credited and that individual graphics may be subject to special legal provisions.
The license is subject to the *Beilstein Journal of Organic Chemistry* terms and conditions: (https://www.beilstein-journals.org/bjoc/terms)

Table of contents

Figures S1-S2 (−)-HRESIMS and ^{1}H NMR spectra of **1** S3
Figures S3-S4 HSQC and COSY NMR spectra of **1** S4
Figures S5-S6 HMBC of 1 and (+)-HRESIMS spectra of 2..S5
Figures S7-S8 ^{1}H NMR and HSQC spectra of **2** S6
Figure S9- COSY spectrum of **2** S7
Figures S10-S11 HMBC of **2** and (-)-HRESIMS spectra of **3**.......................................S8
Figures S12-S13 ^{1}H NMR and HSQC spectra of **3** S9
Figures S14-S15 COSY and HMBC spectra of **3**...S10
Figures S16-S17 NOESY and ^{13}C NMR spectra of **3**..S11
Figures S18-S19 (−)-HRESIMS and ^{1}H NMR spectra of **4**...S12
Figures S20-S21 HSQC and COSY NMR spectra of **4** S13
Figures S22-S23 HMBC and NOESY spectra of **4** S14
Figures S24-S25 (+)-HRESIMS and ^{1}H NMR spectra of **5**……………………………...S15
Figures S26-S27 HSQC and COSY NMR spectra of **5** S16
Figures S28-S29 HMBC of **5** and (+)-HRESIMS spectra of **6**......................................S17
Figures S30-S31 ^{1}H NMR and HSQC spectra of **6**..S18
Figures S32-S33 COSY and HMBC spectra of **6**...S19
Figures S34-S35 (−)-HRESIMS and ^{1}H NMR spectra of **7**...S20
Figures S36-S37 HSQC and COSY NMR spectra of **7**..S21
Figure S36 HMBC spectrum of **7**…………………..S22
Figure S37. HPLC profiles of L-FDAA derivatised amino acid standards and L-FDAA derivatised hydrolysates of compounds **1–3** and Marfey's analysis results………………………………………………………………………………………S22
Table S1. In-vitro activities of compounds **1–4** and cytotoxicity assay………………...S24
References…………………………………………………………………………………S24

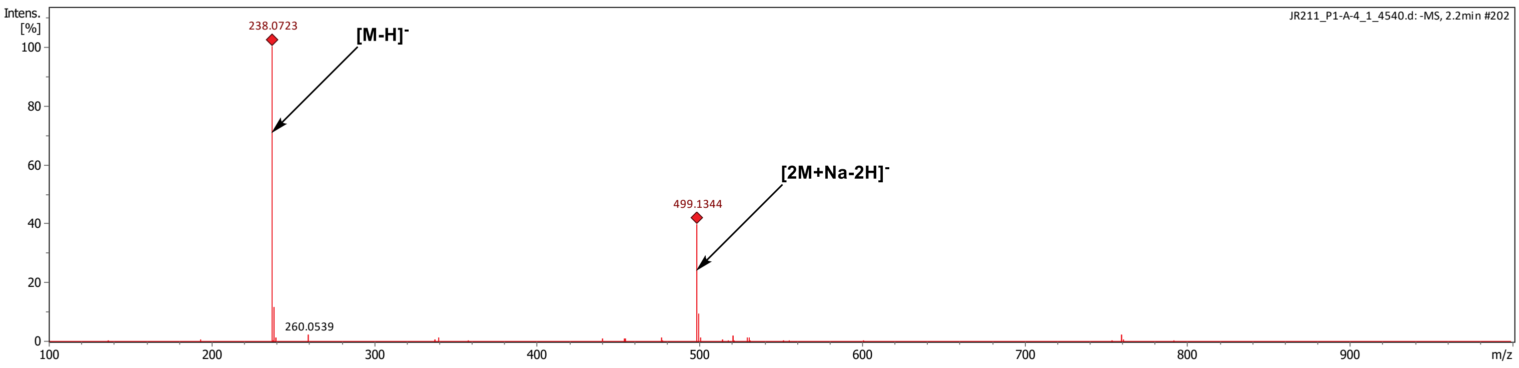

Figure S1. (–)-HR-ESIMS spectrum of **1**.

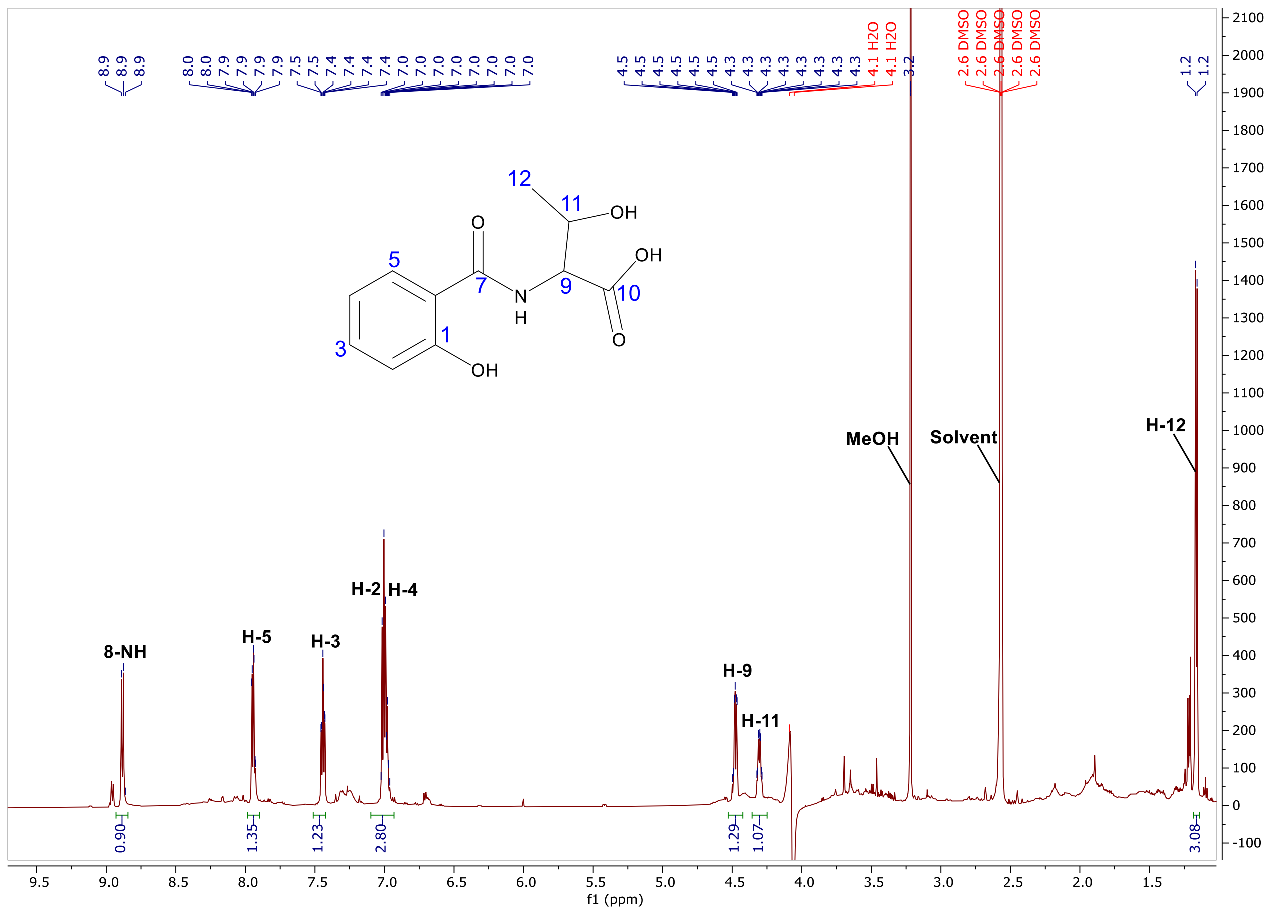

Figure S2. ^{1}H NMR spectrum of **1**.

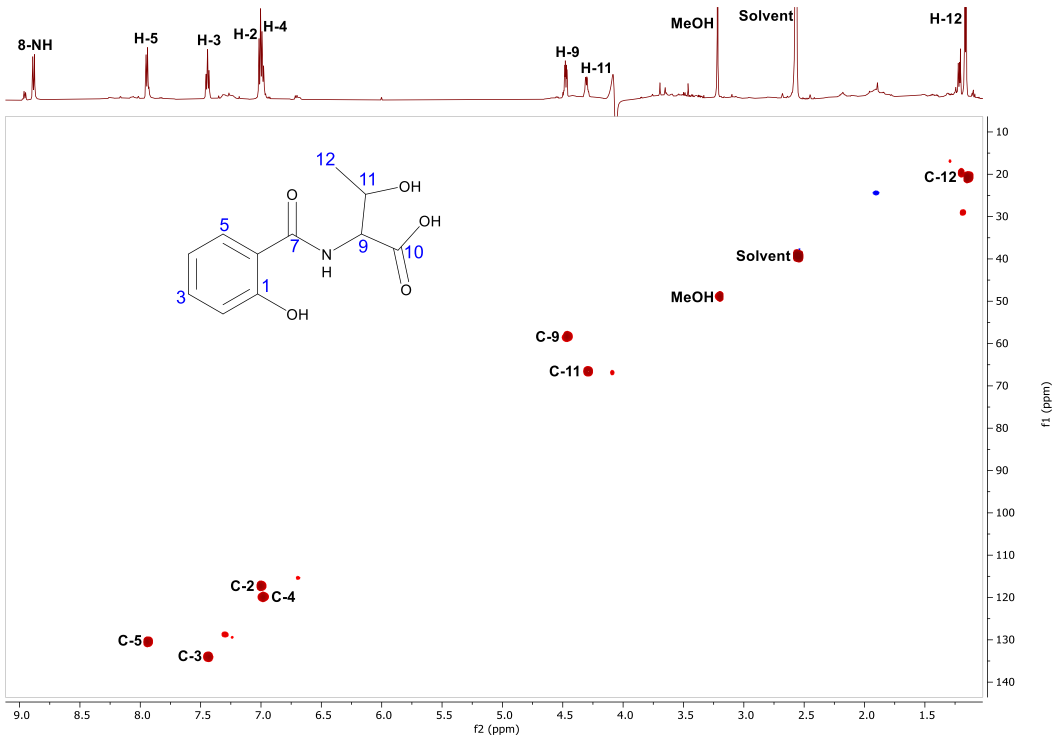

Figure S3. HSQC spectrum of **1**.

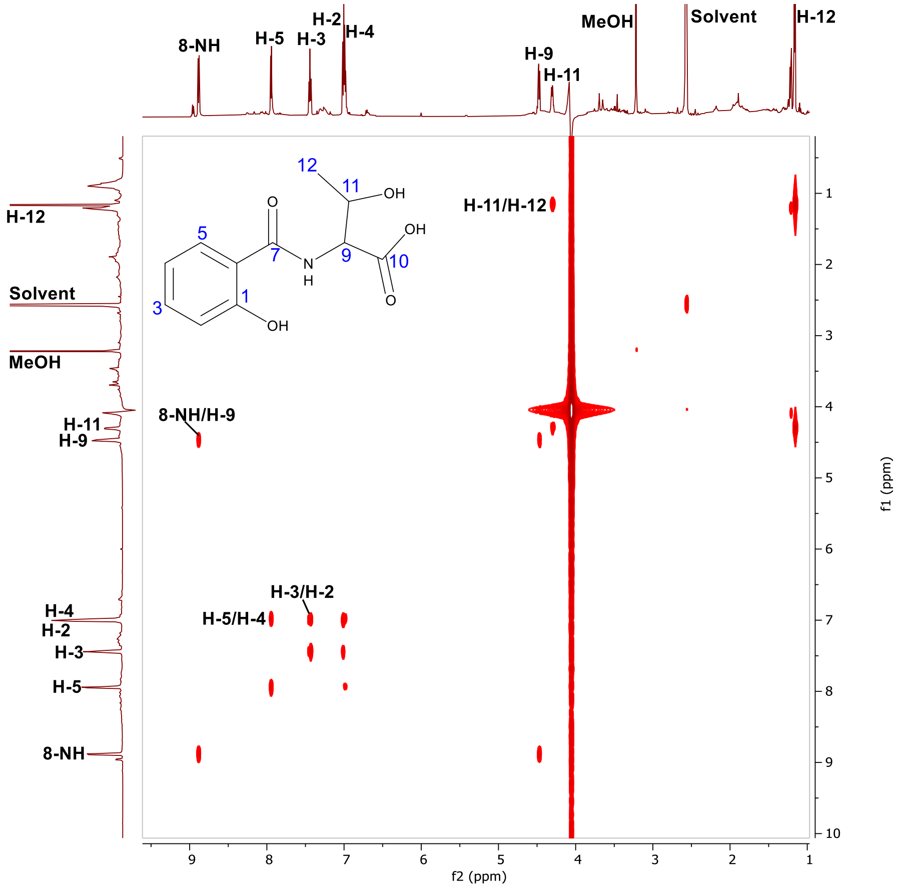

Figure S4. COSY spectrum of **1**.

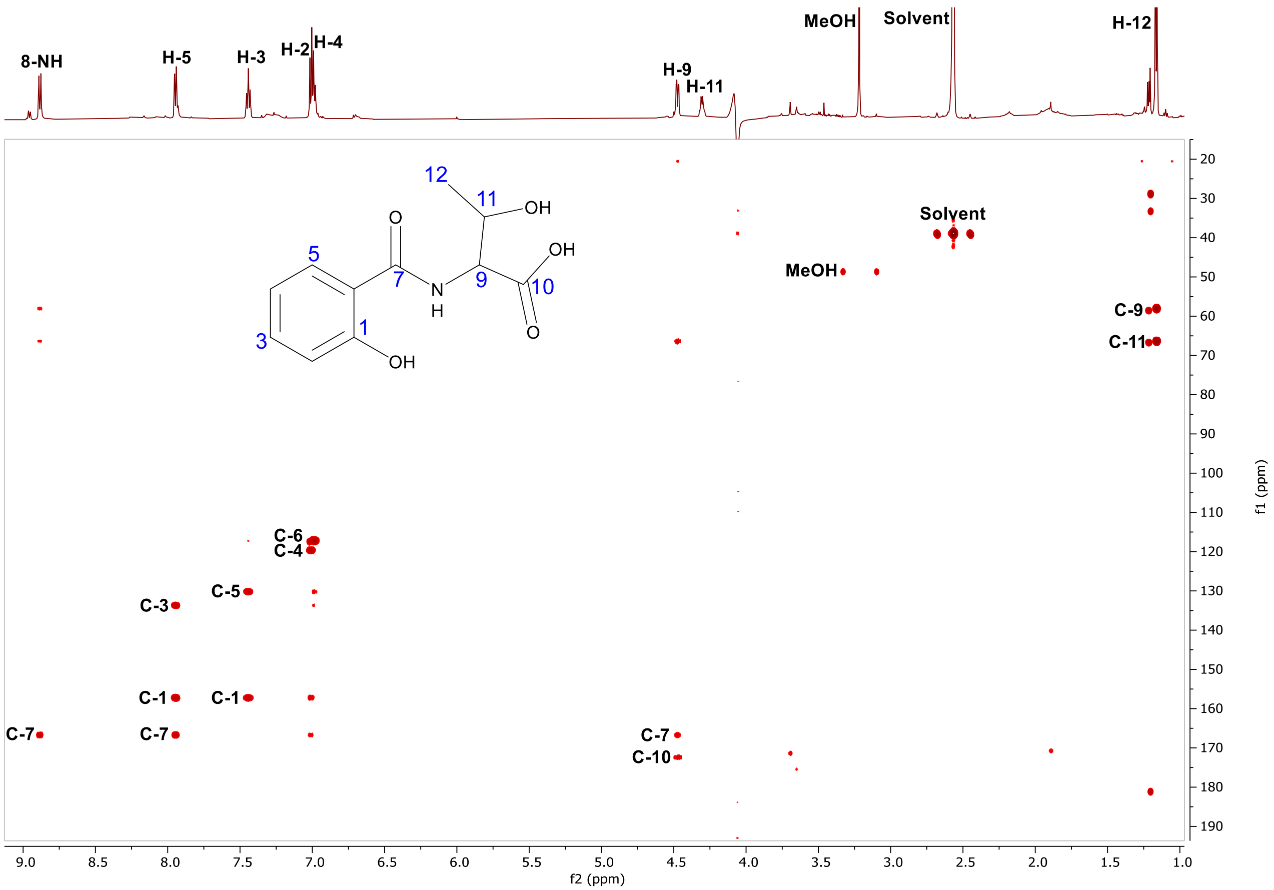

Figure S5. HMBC spectrum of **1**.

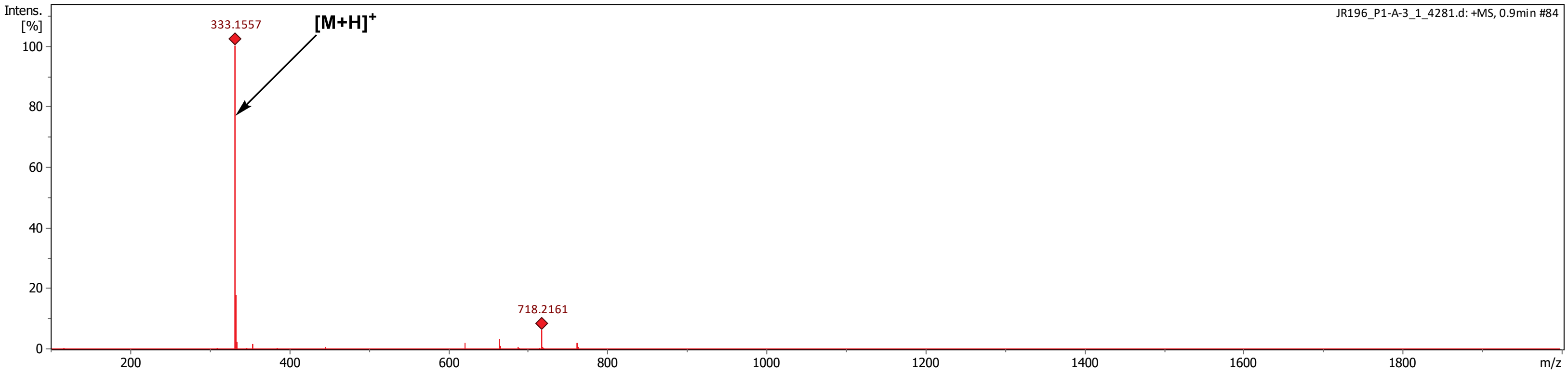

Figure S6. (+)-HR-ESIMS spectrum of **2**.

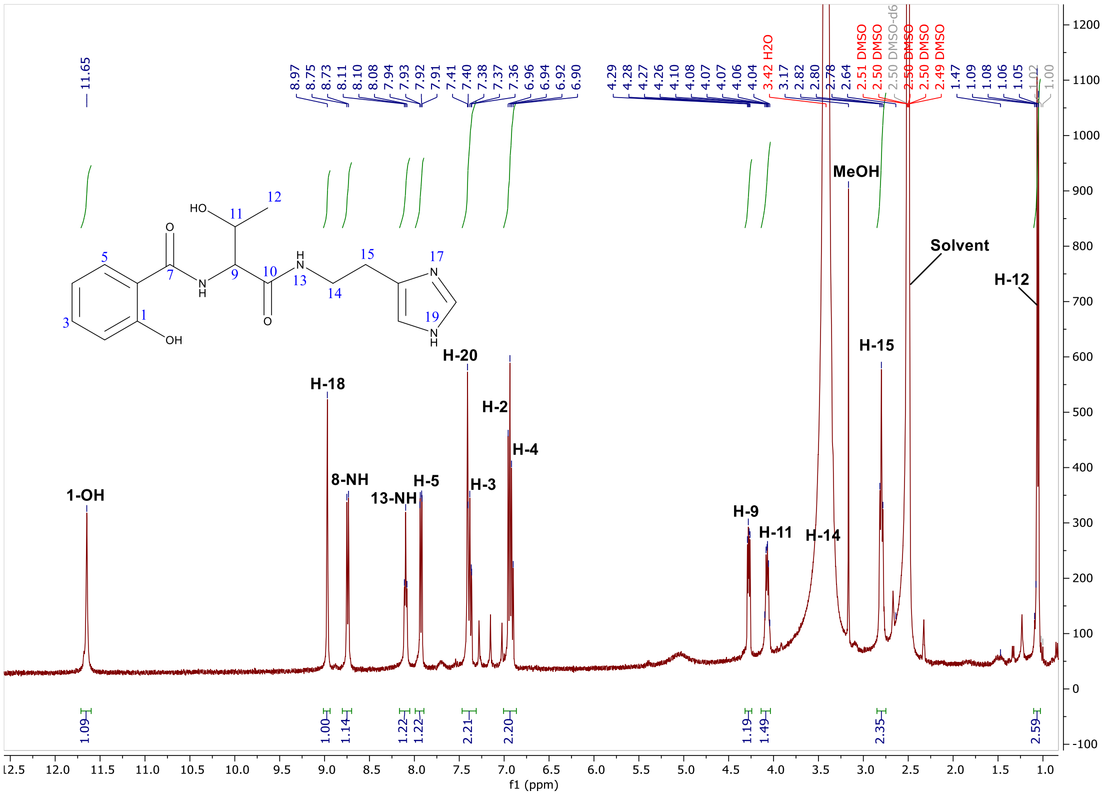

Figure S7. ^{1}H NMR spectrum of **2**.

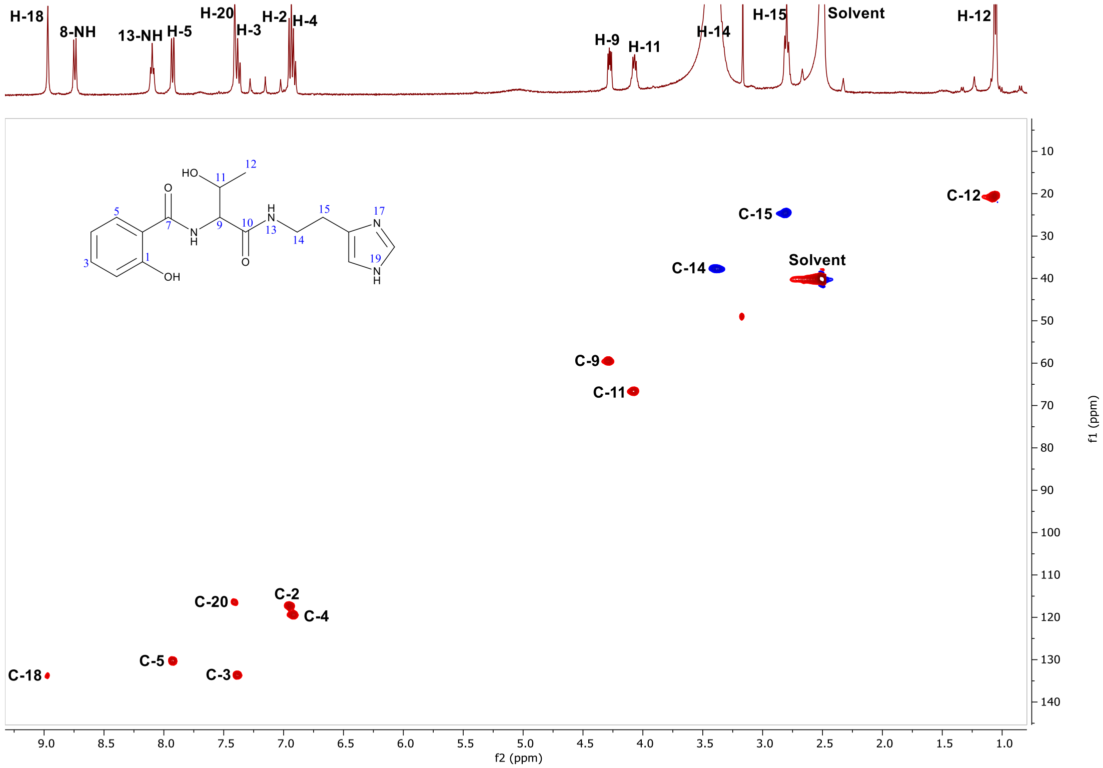

Figure S8. HSQC spectrum of **2**

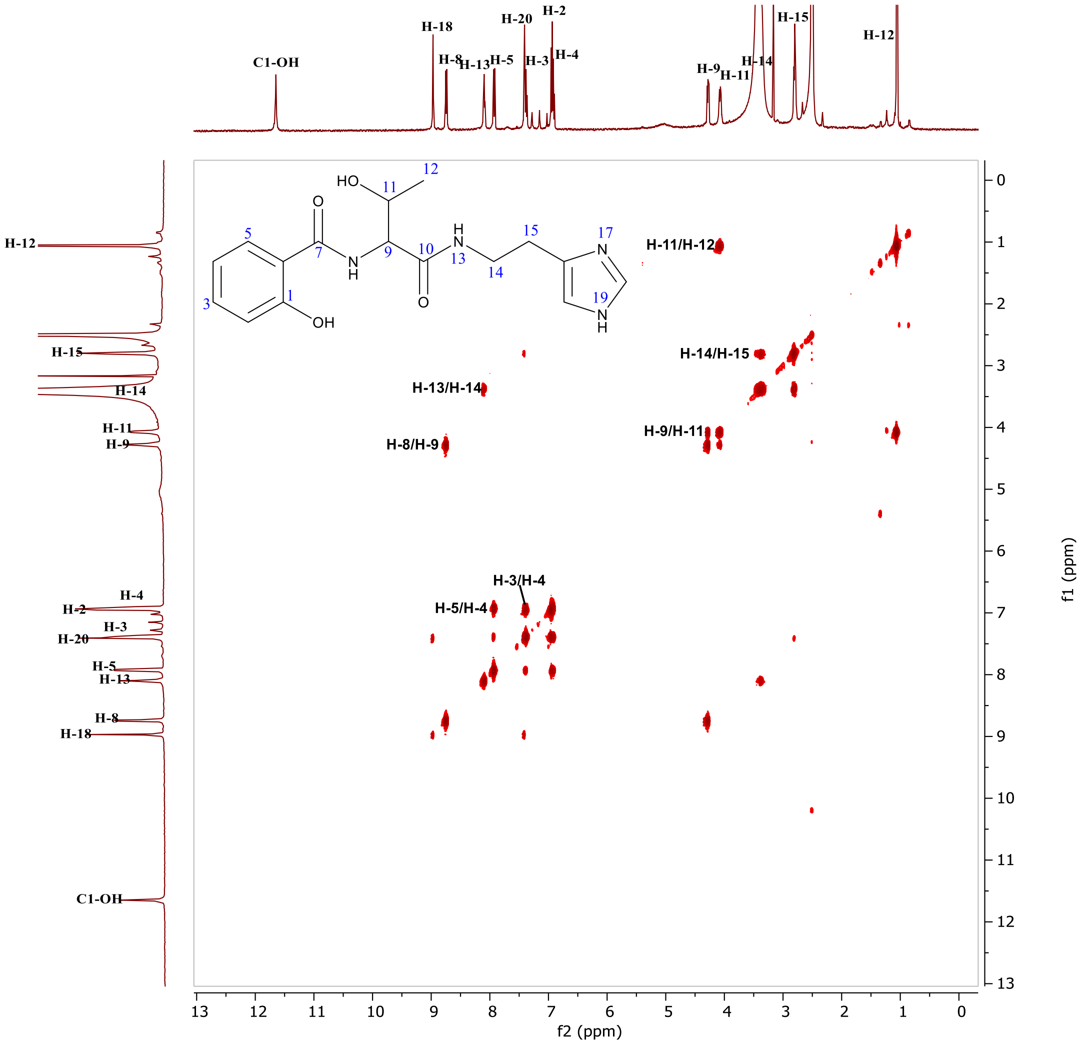

Figure S9. COSY spectrum of **2**.

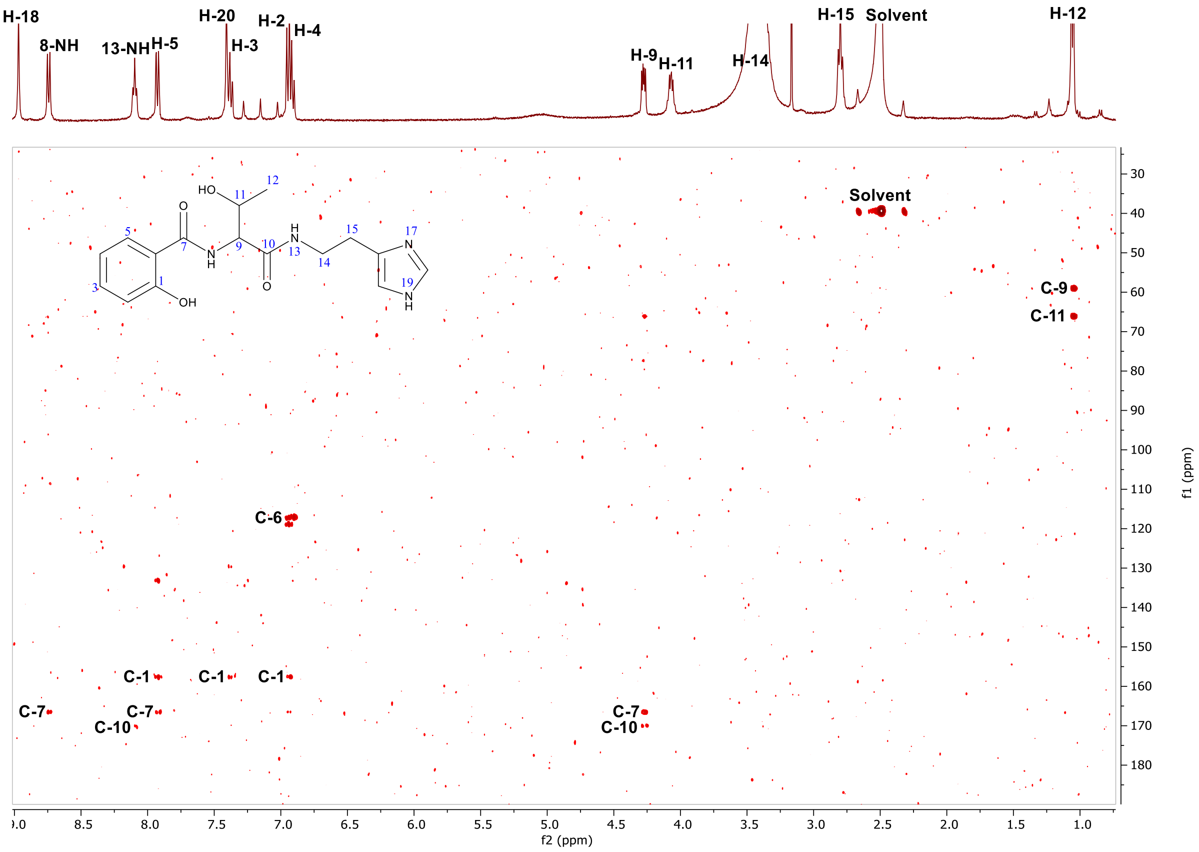

Figure S10. HMBC spectrum of **2**.

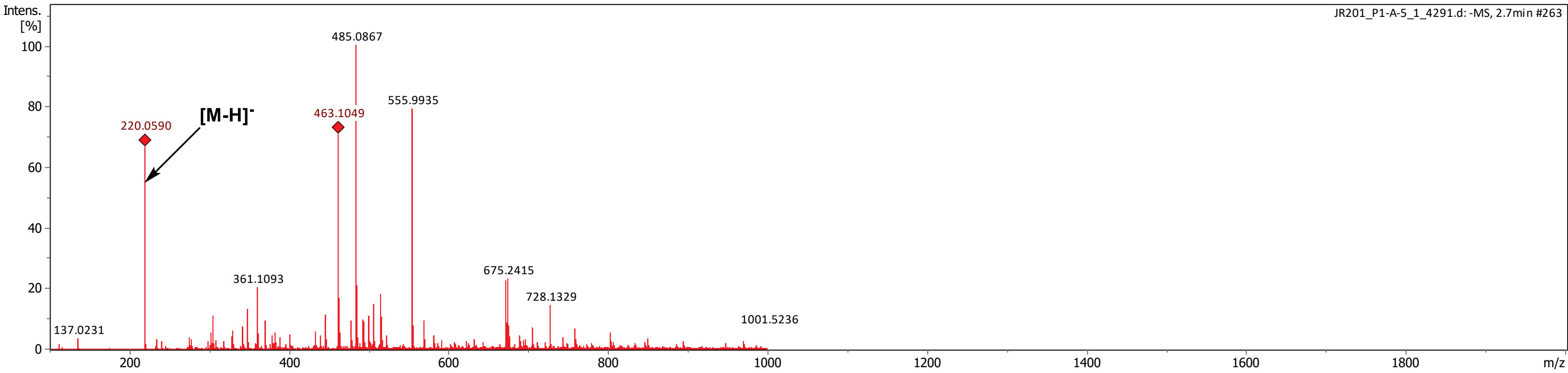

Figure S11. (-)-HR-ESIMS spectrum of **3**.

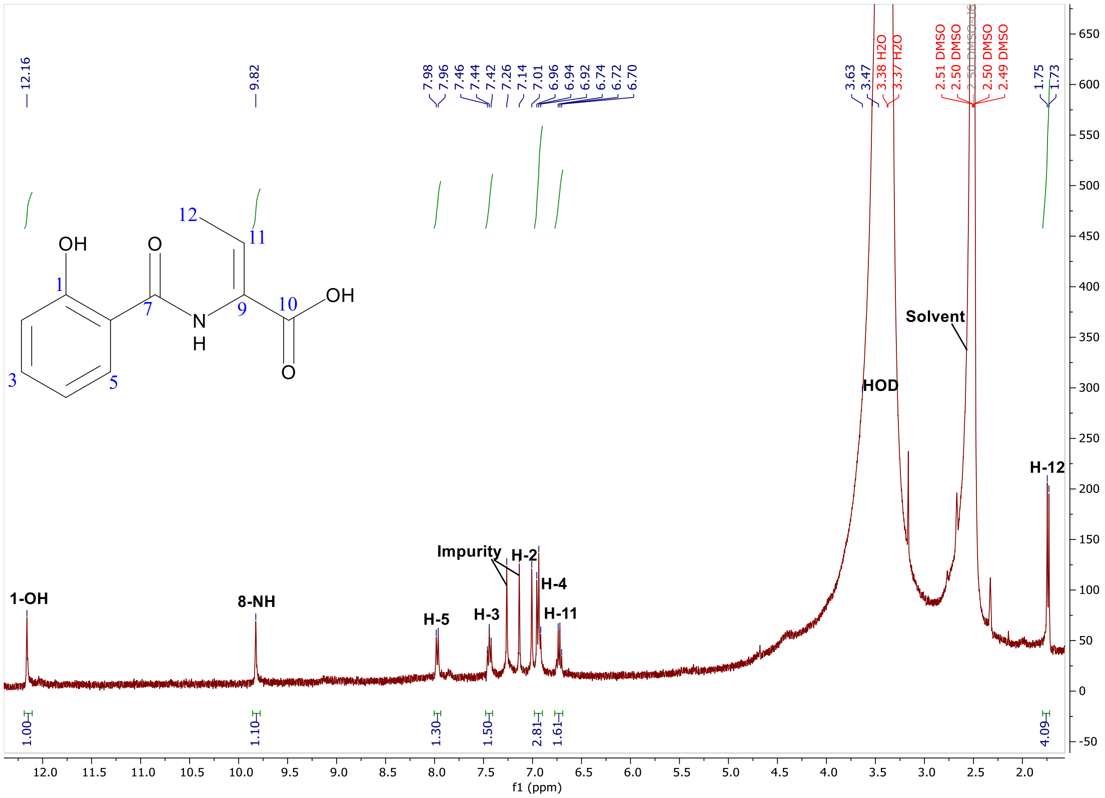

Figure S12. ^{1}H NMR spectrum of **3**.

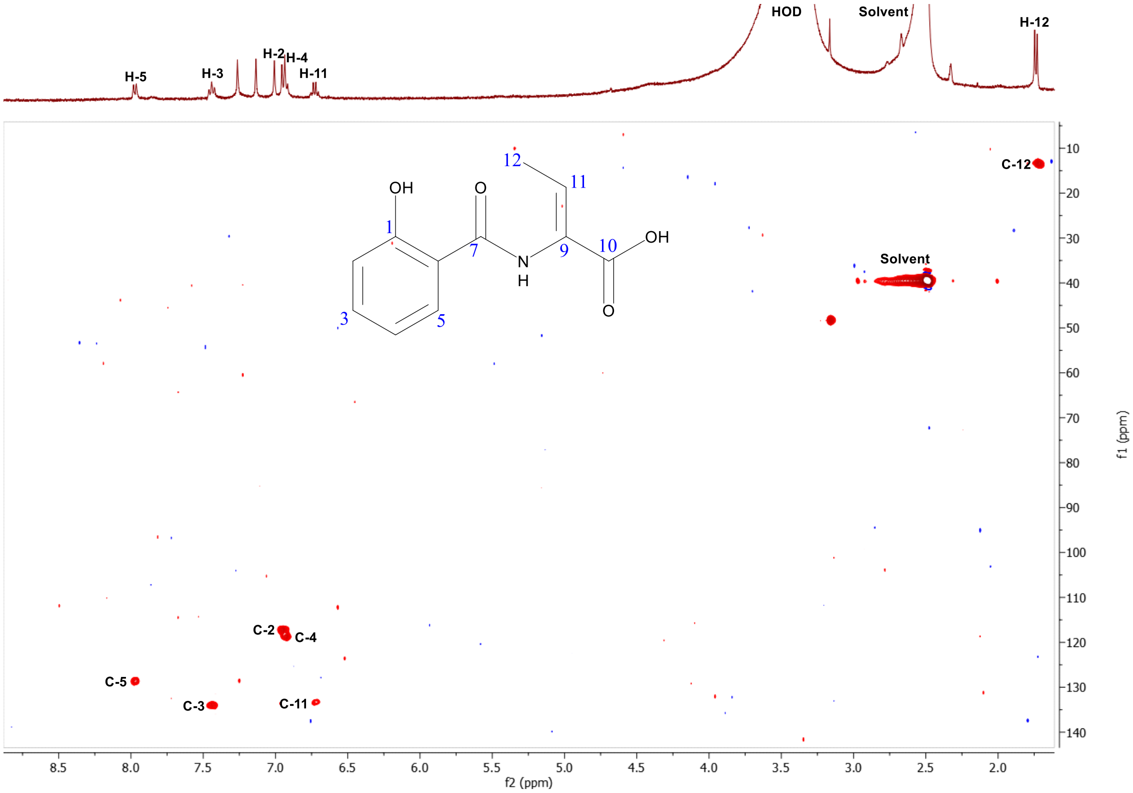

Figure S13. HSQC spectrum of **3**.

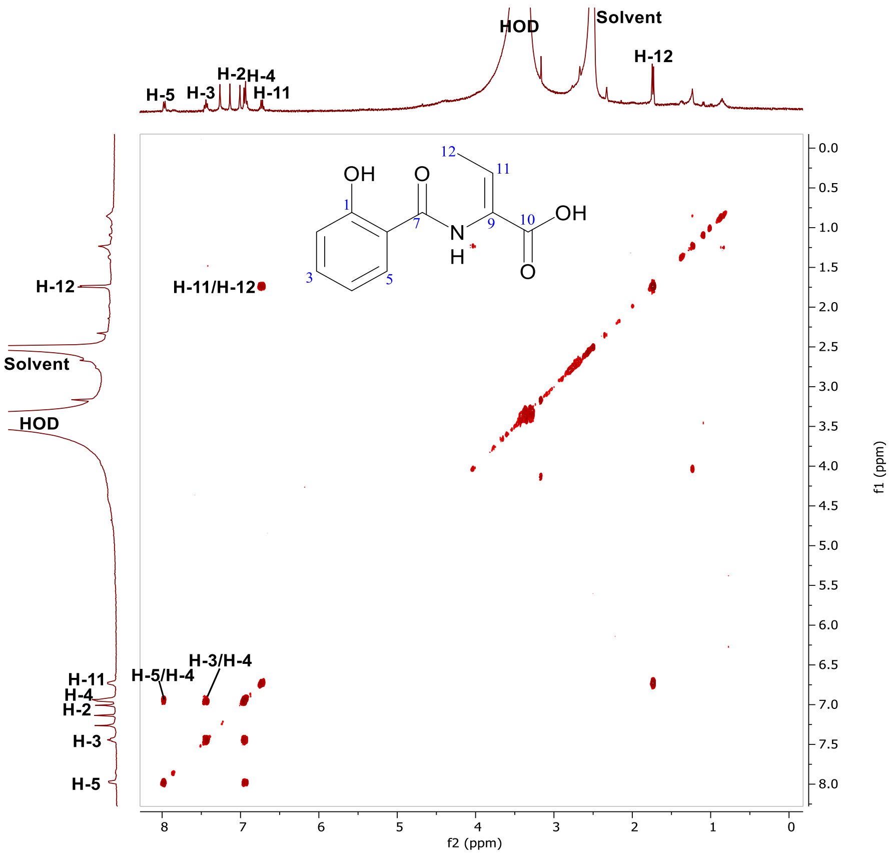

Figure S14. COSY spectrum of **3.**

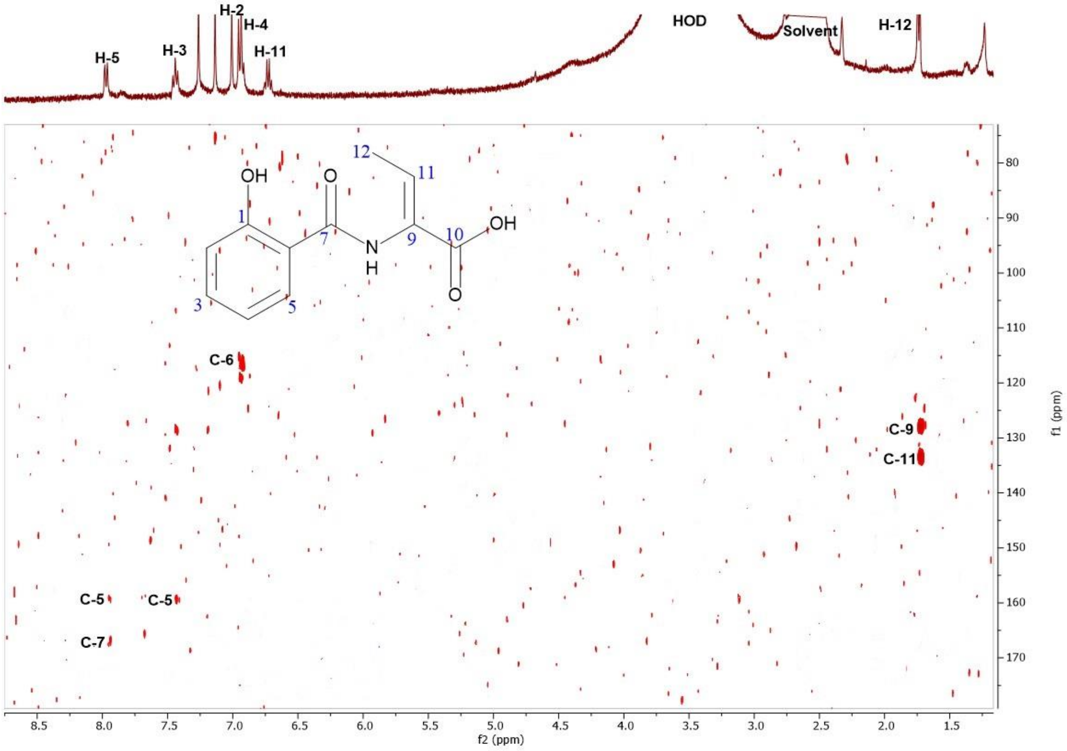

Figure S15. HMBC spectrum of **3**.

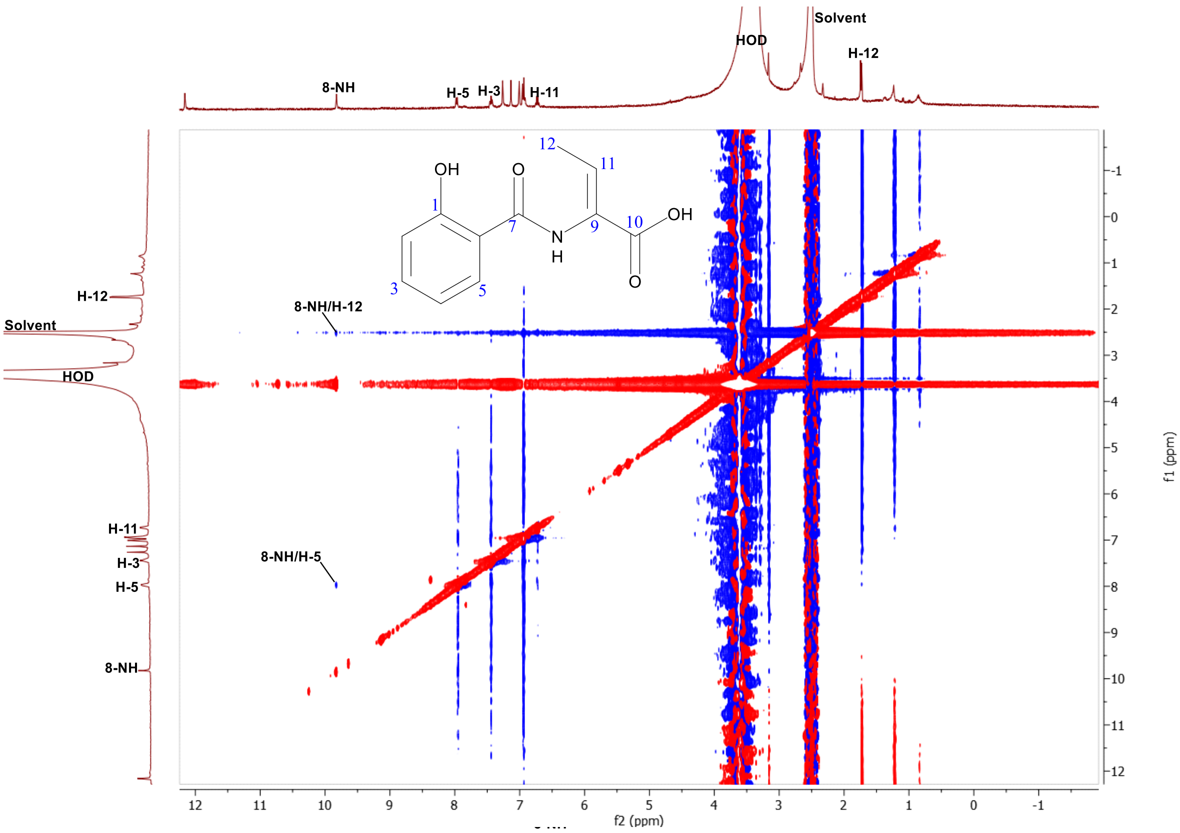

Figure S16. NOESY spectrum of **3**.

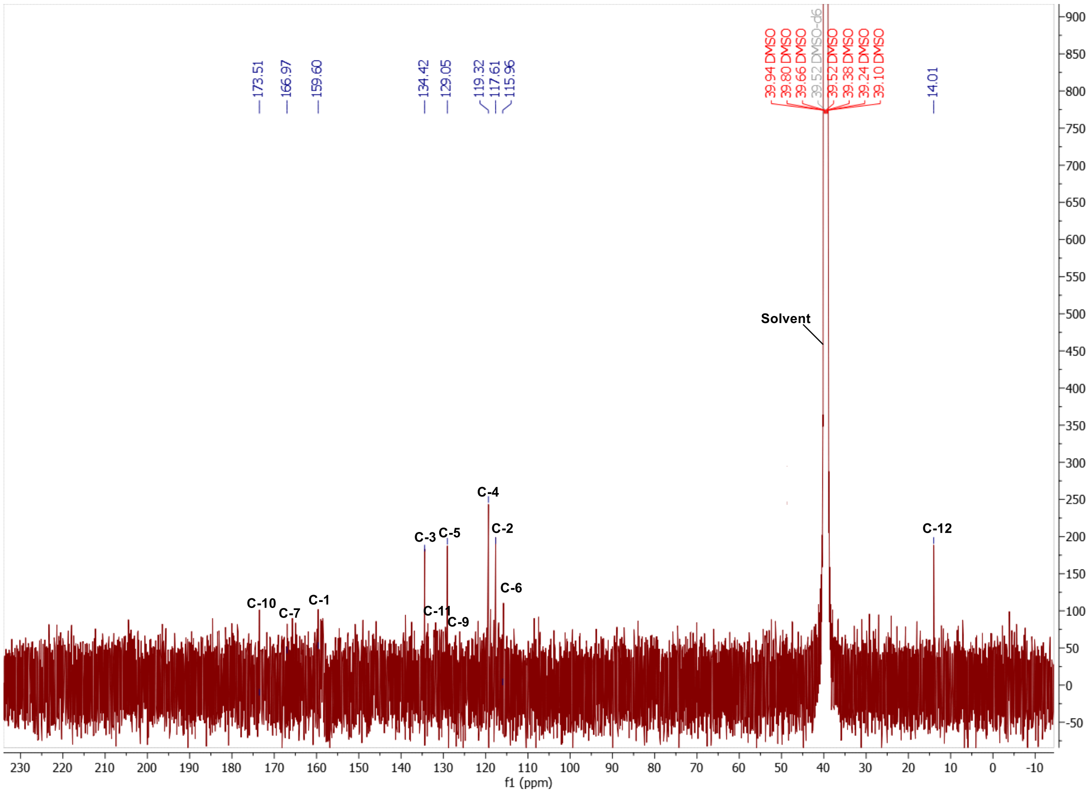

Figure S17. ^{13}C NMR spectrum of **3**.

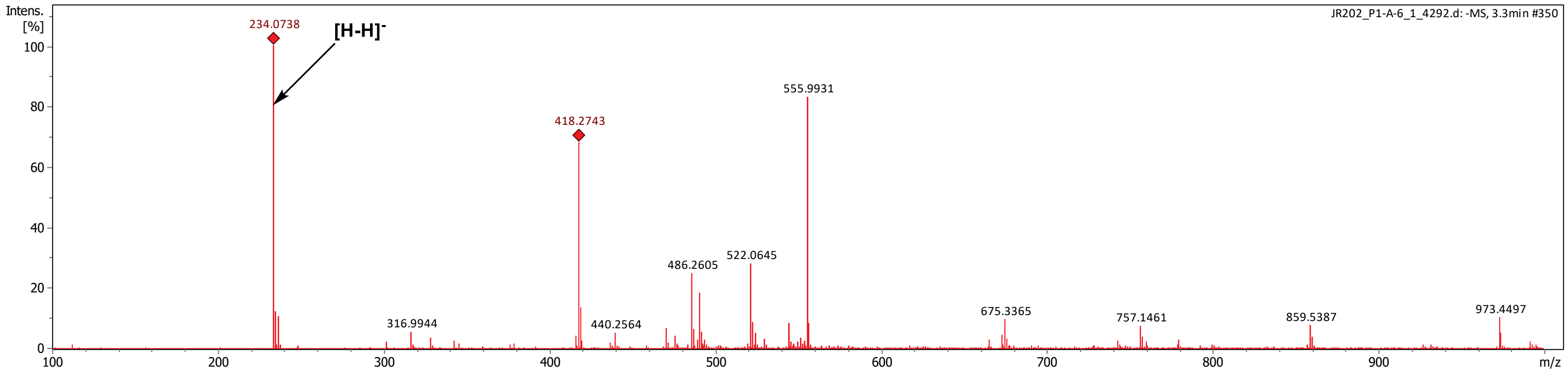

Figure S18. (–)-HRESIMS spectrum of **4**.

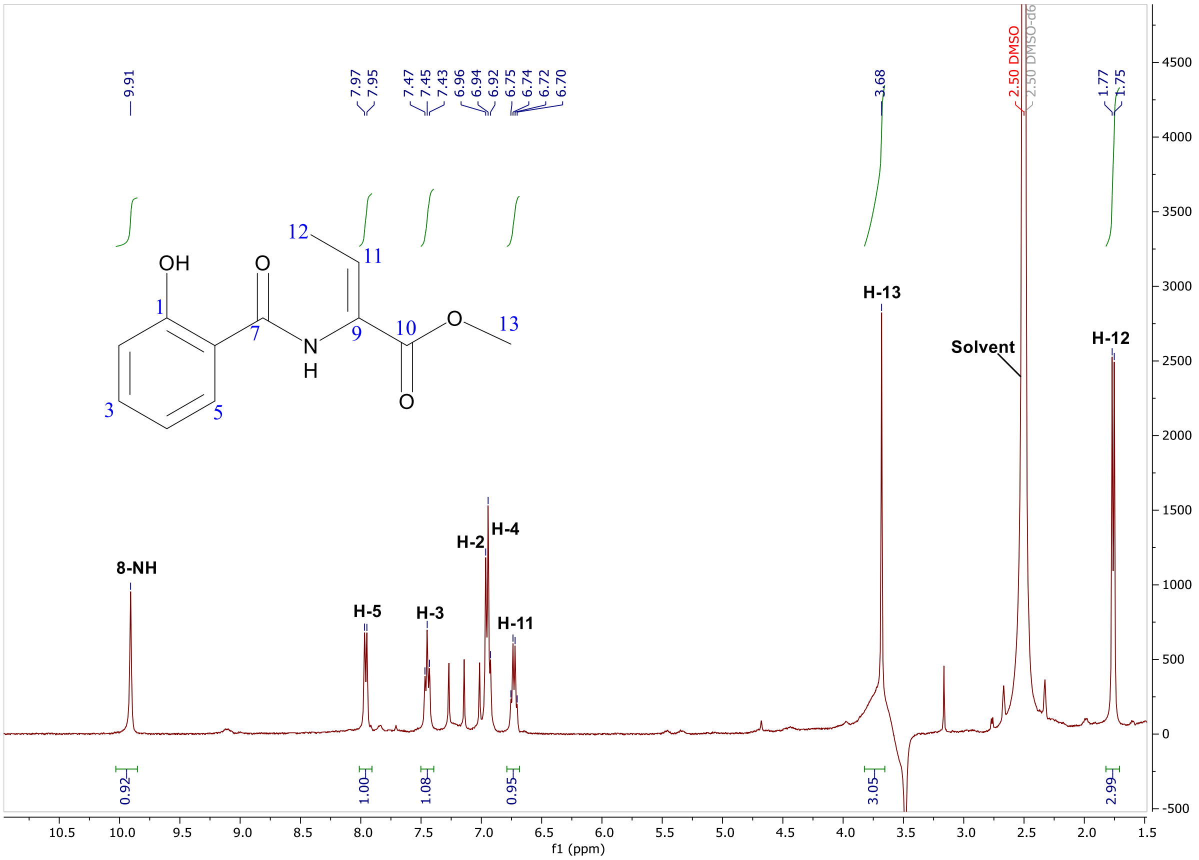

Figure S19. ^{1}H NMR spectrum of **4**.

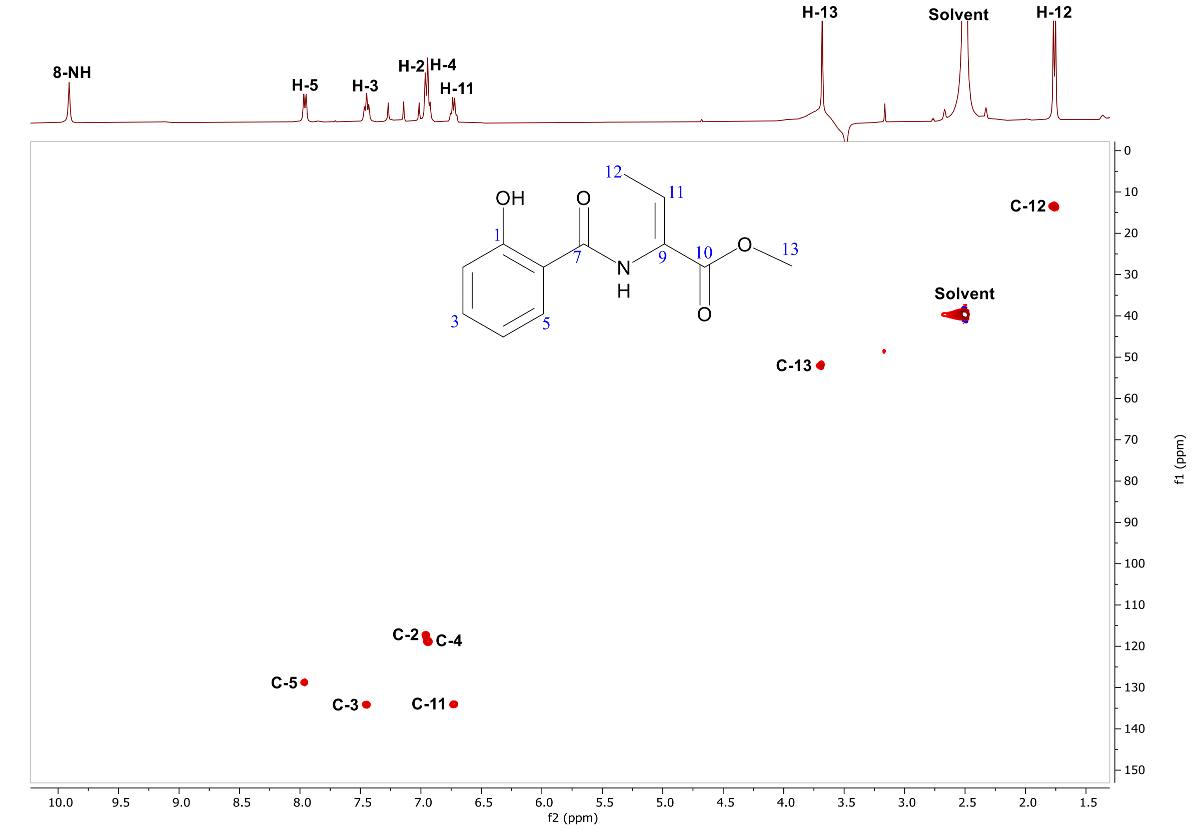

Figure S20. HSQC spectrum of **4**.

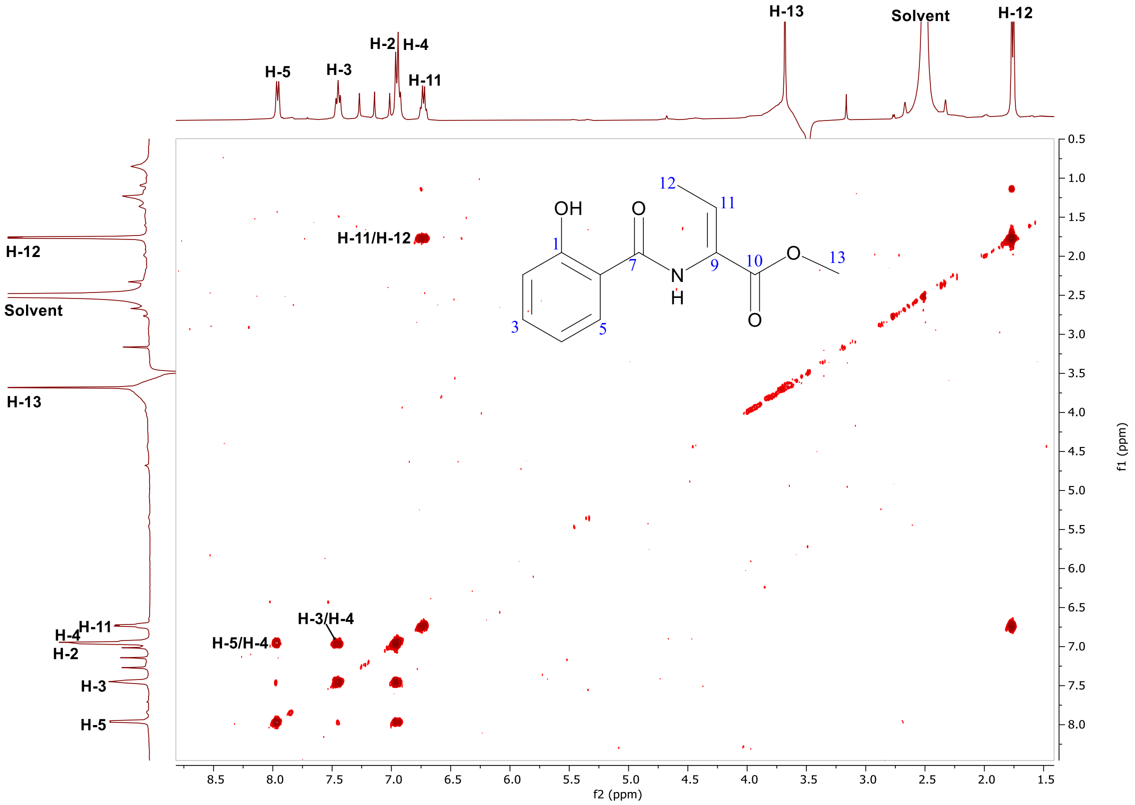

Figure S21. COSY spectrum of **4**.

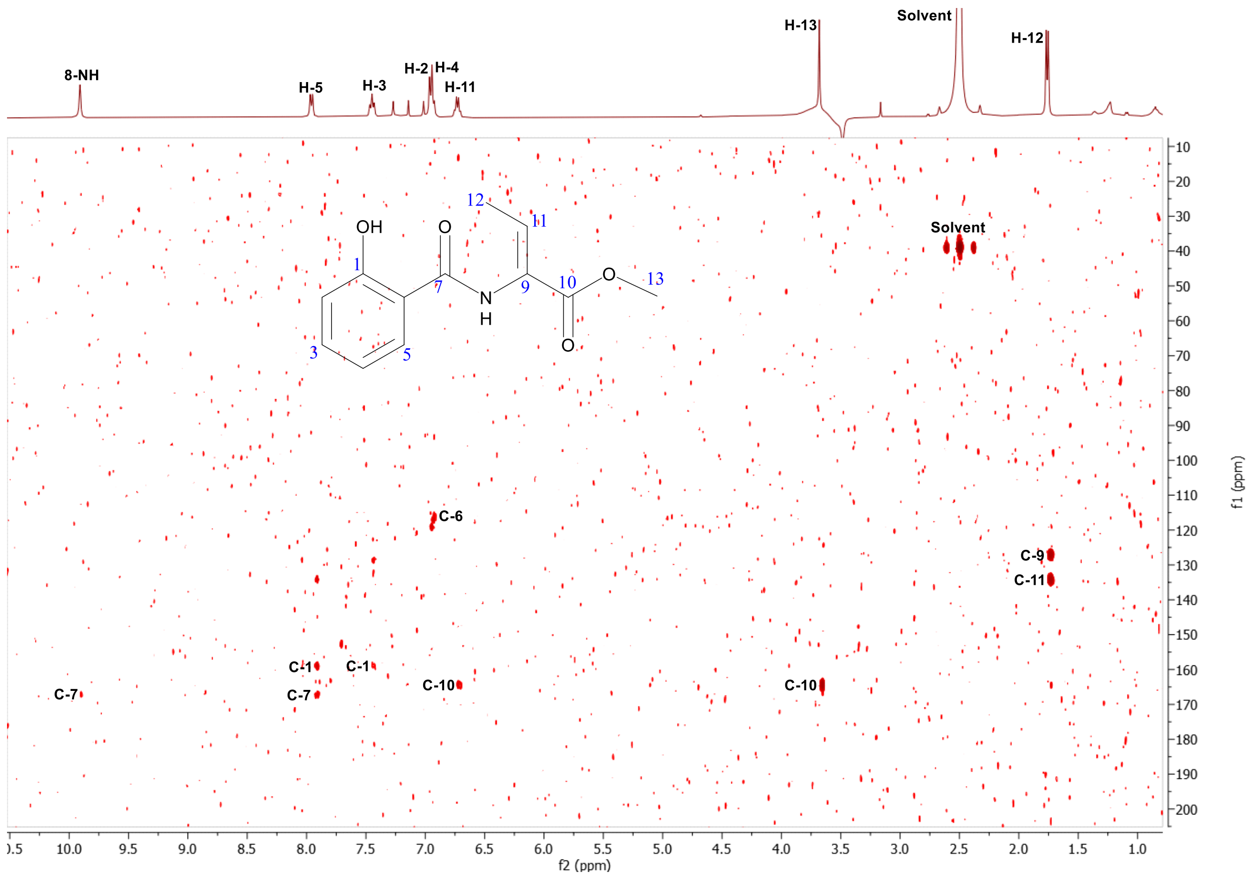

Figure S22. HMBC spectrum of **4**.

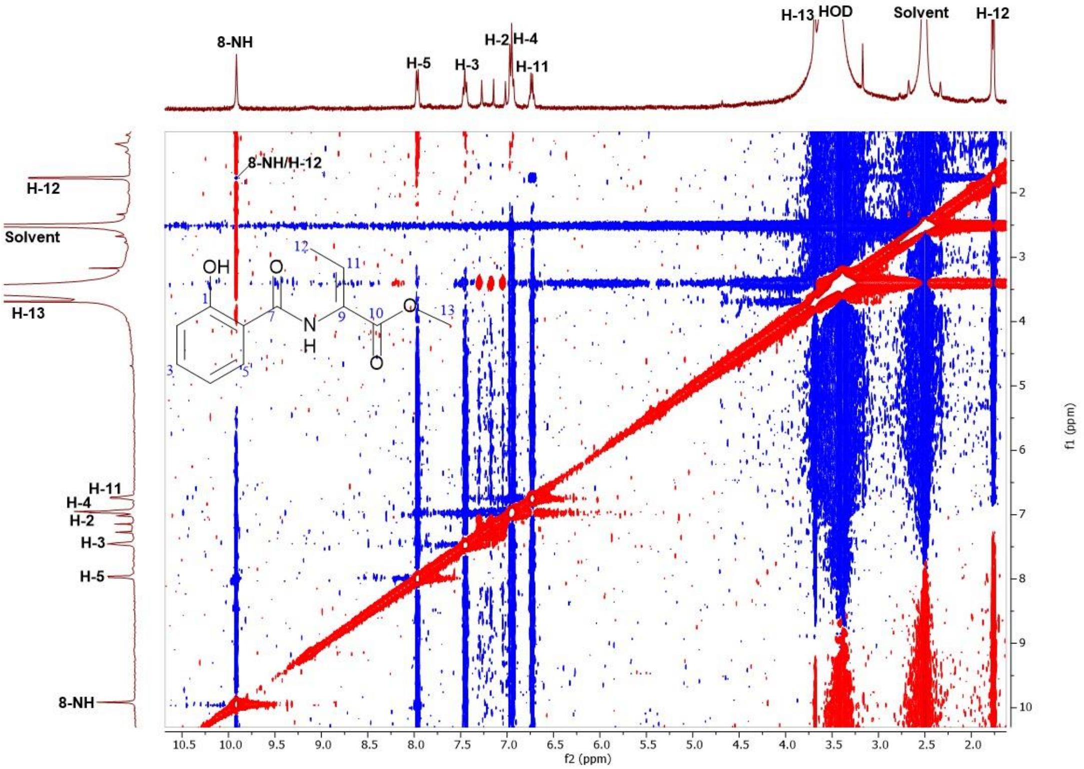

Figure S23. NOESY spectrum of **4**.

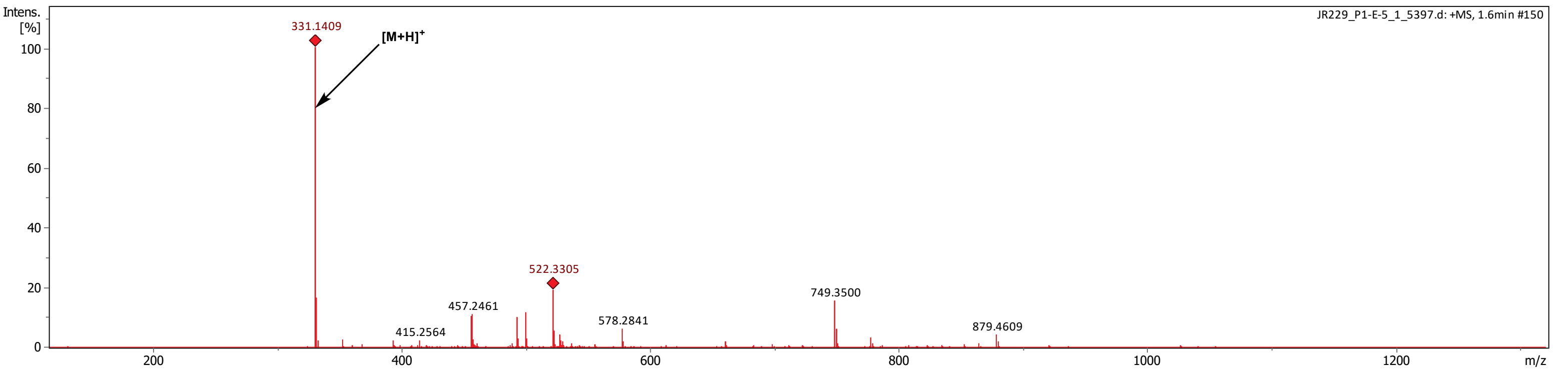

Figure S24. (+)-HRESIMS spectrum of **5**.

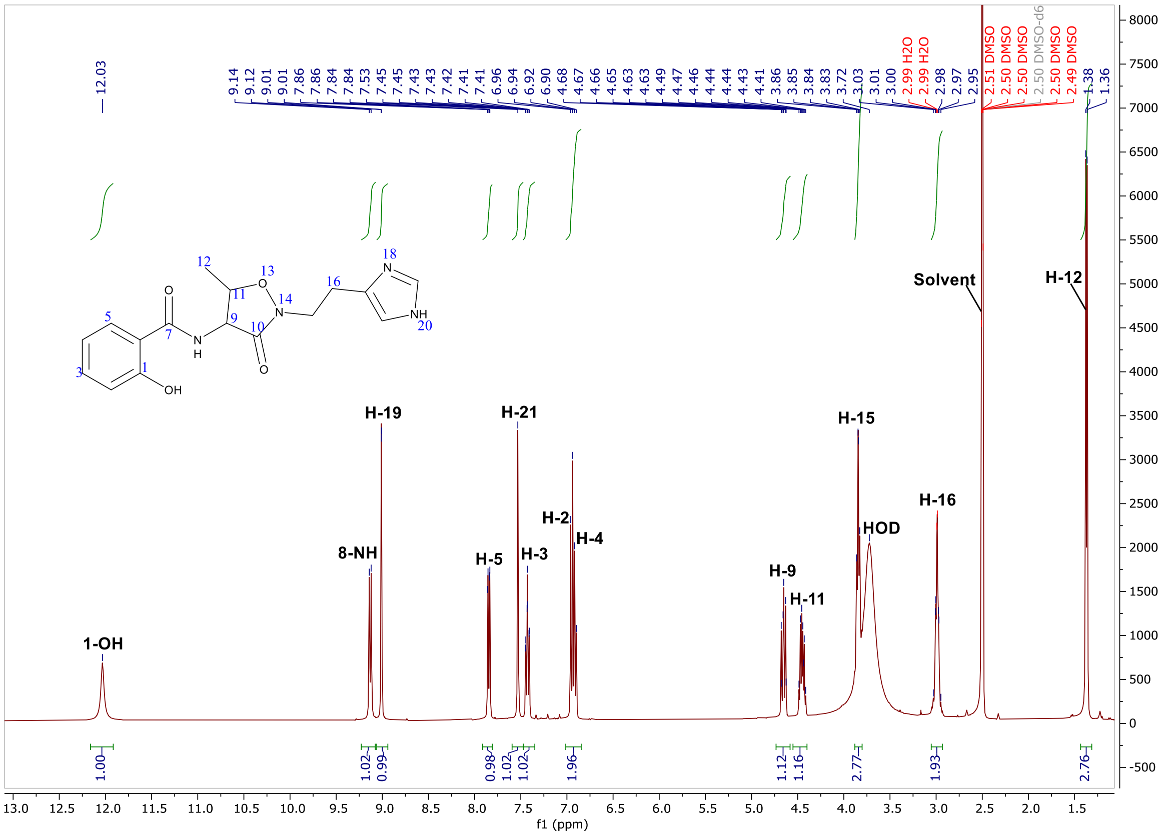

Figure S25. ^{1}H NMR spectrum of **5**.

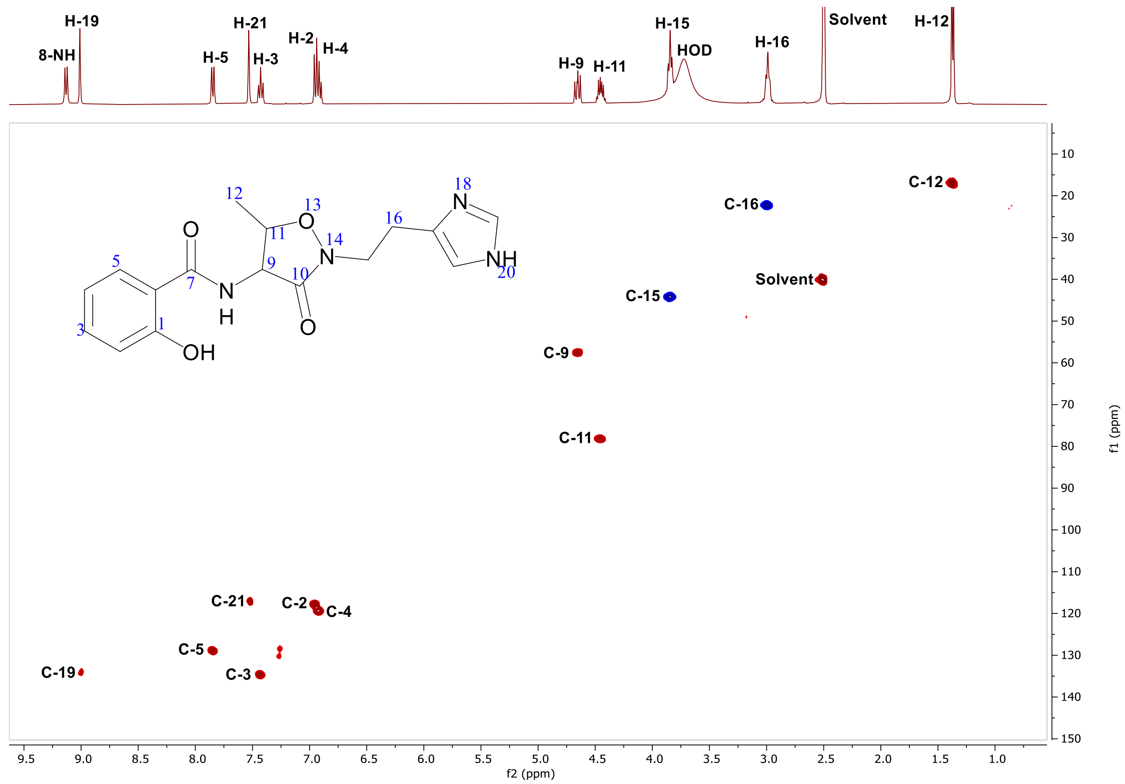

Figure S26. HSQC spectrum of **5**.

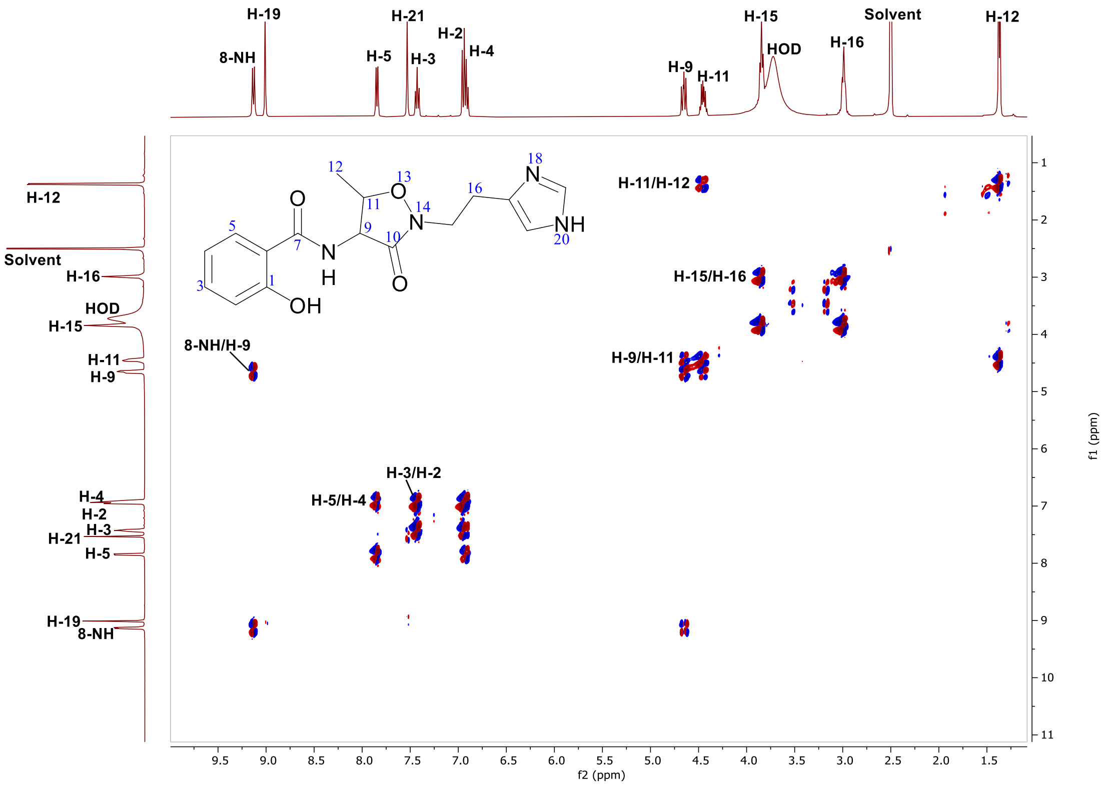

Figure S27. COSY spectrum of **5**.

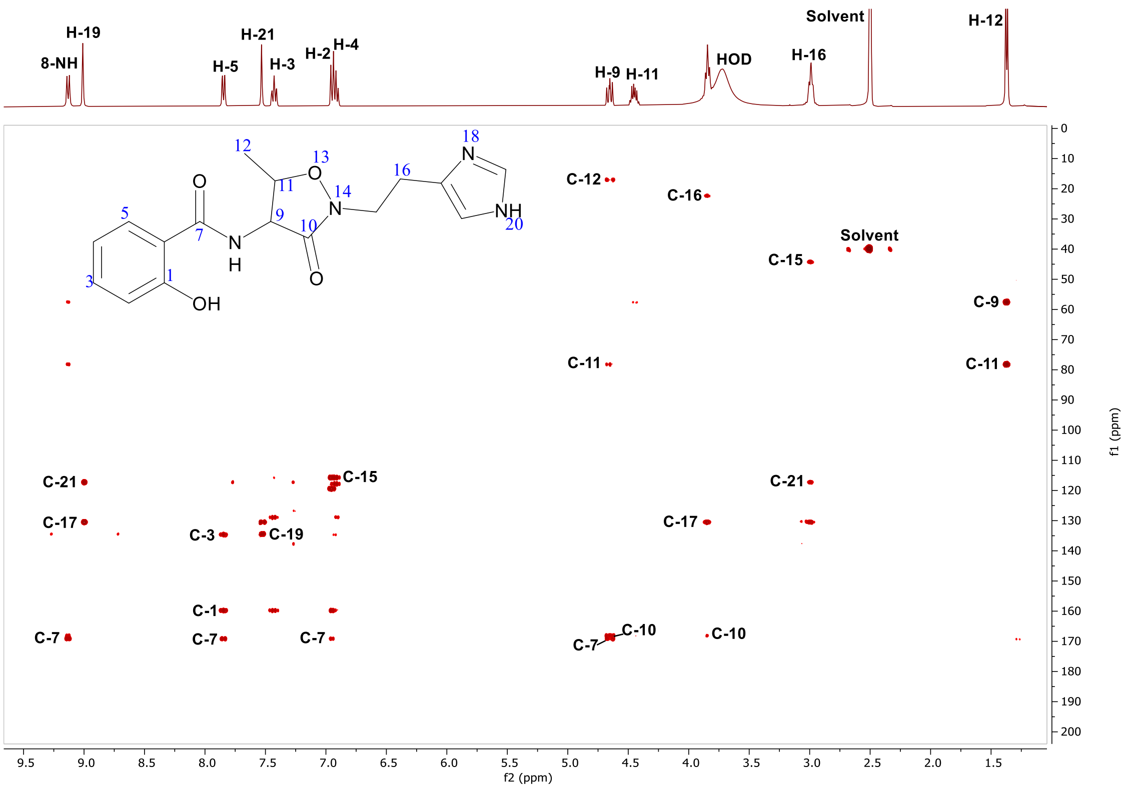

Figure S28. HMBC spectrum of **5**.

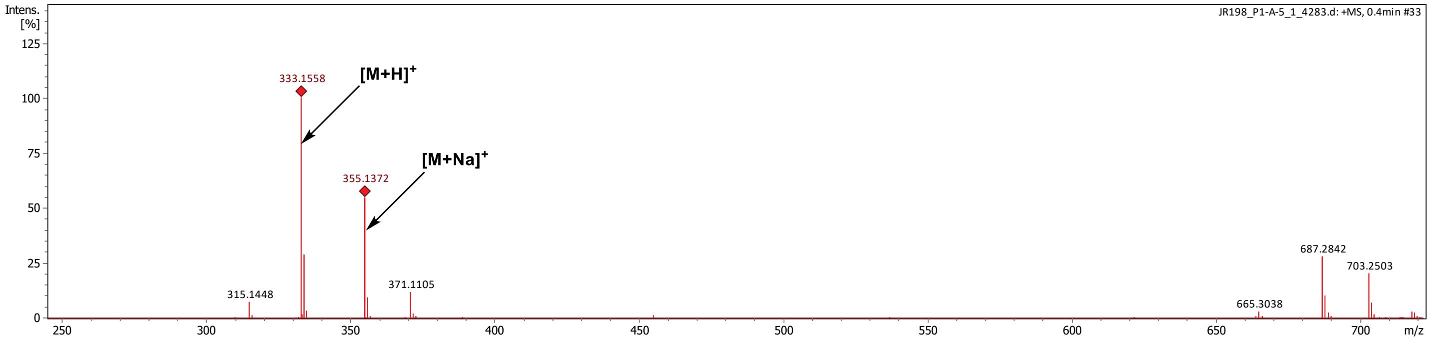

Figure S29. (+)-HRESIMS spectrum of **6**.

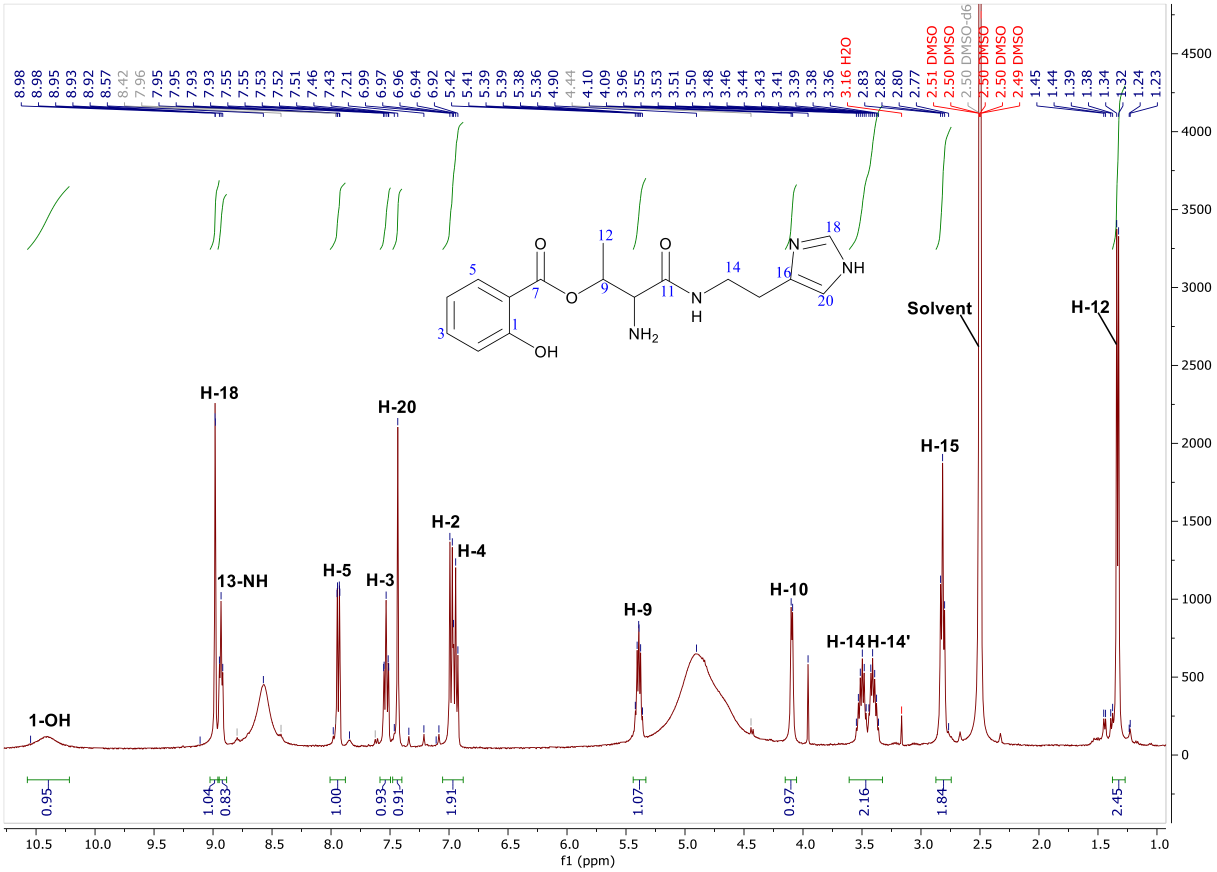

Figure S30. ^{1}H NMR spectrum of **6**.

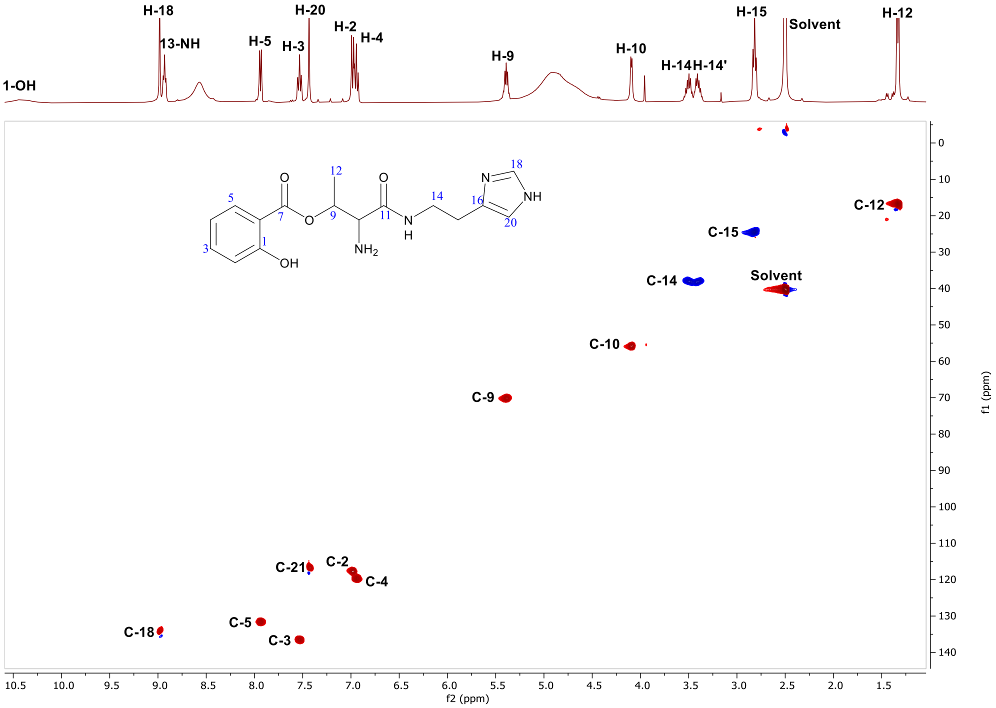

Figure S31. HSQC spectrum of **6.**

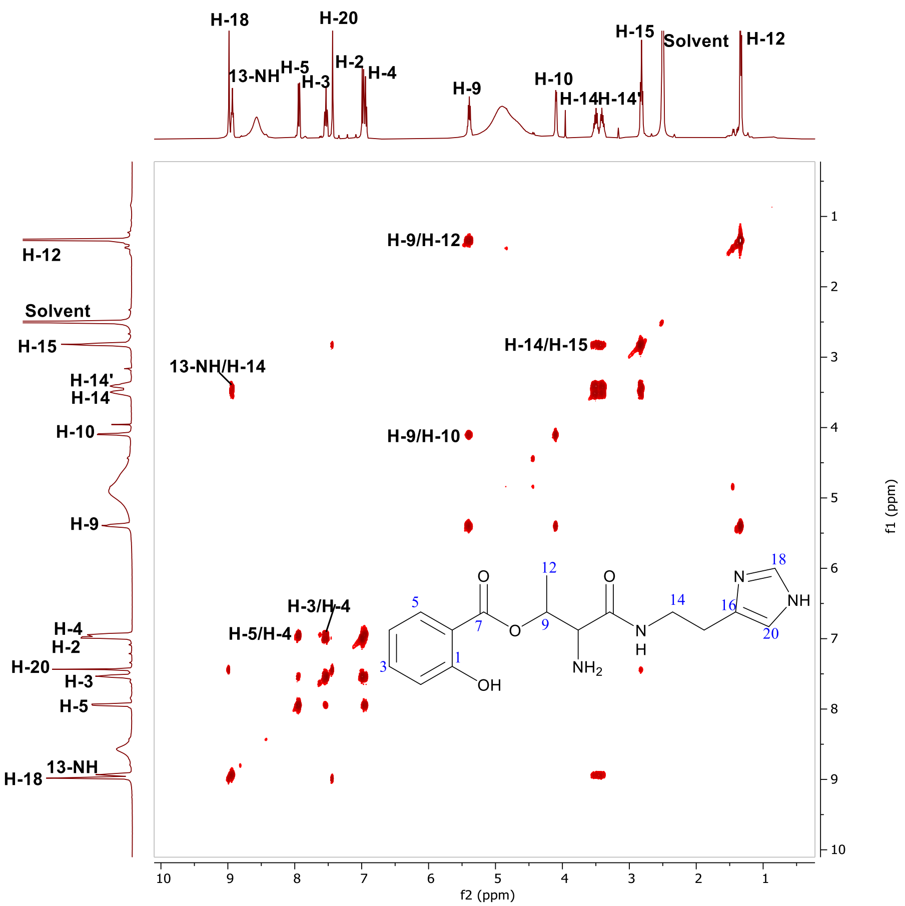

Figure S32. COSY spectrum of **6**.

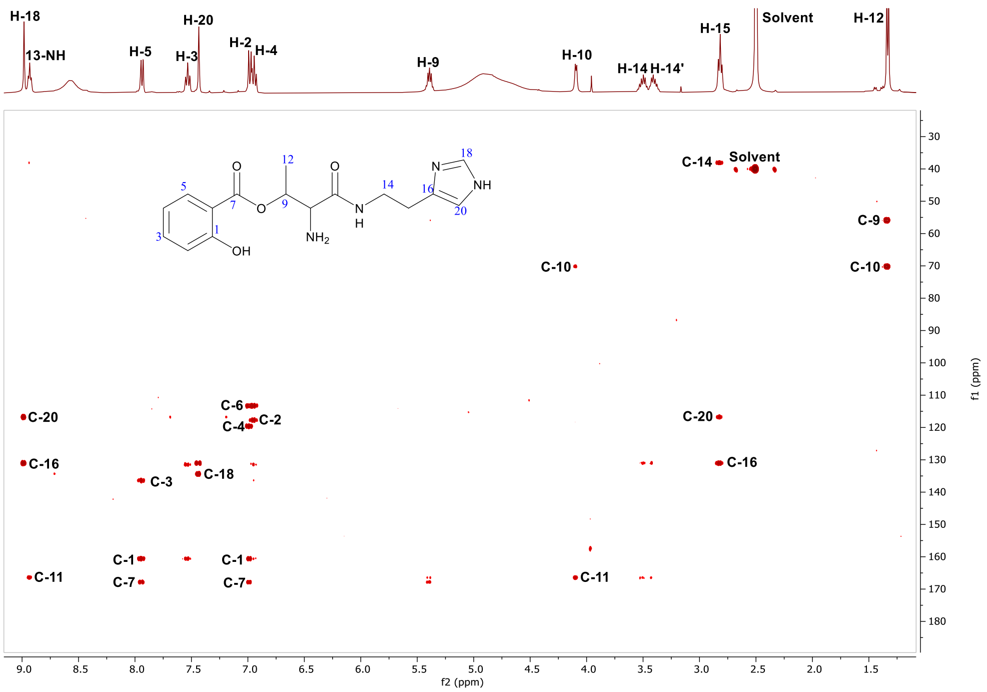

Figure S33. HMBC spectrum of **6**.

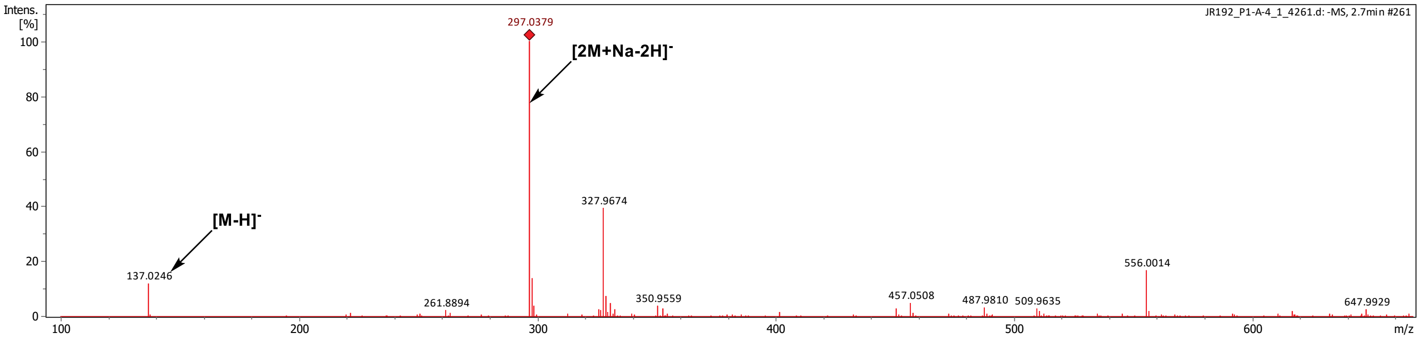

Figure S34. (–)-HRESIMS spectrum of **7.**

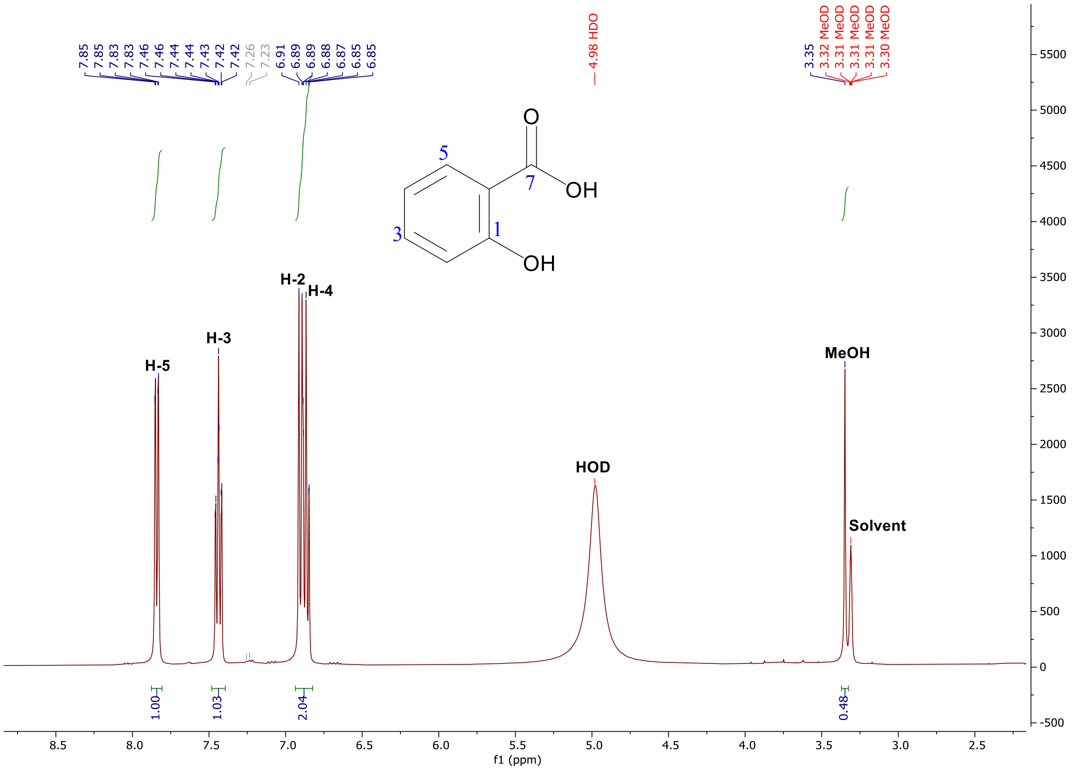

Figure S35. ^{1}H NMR spectrum of **7.**

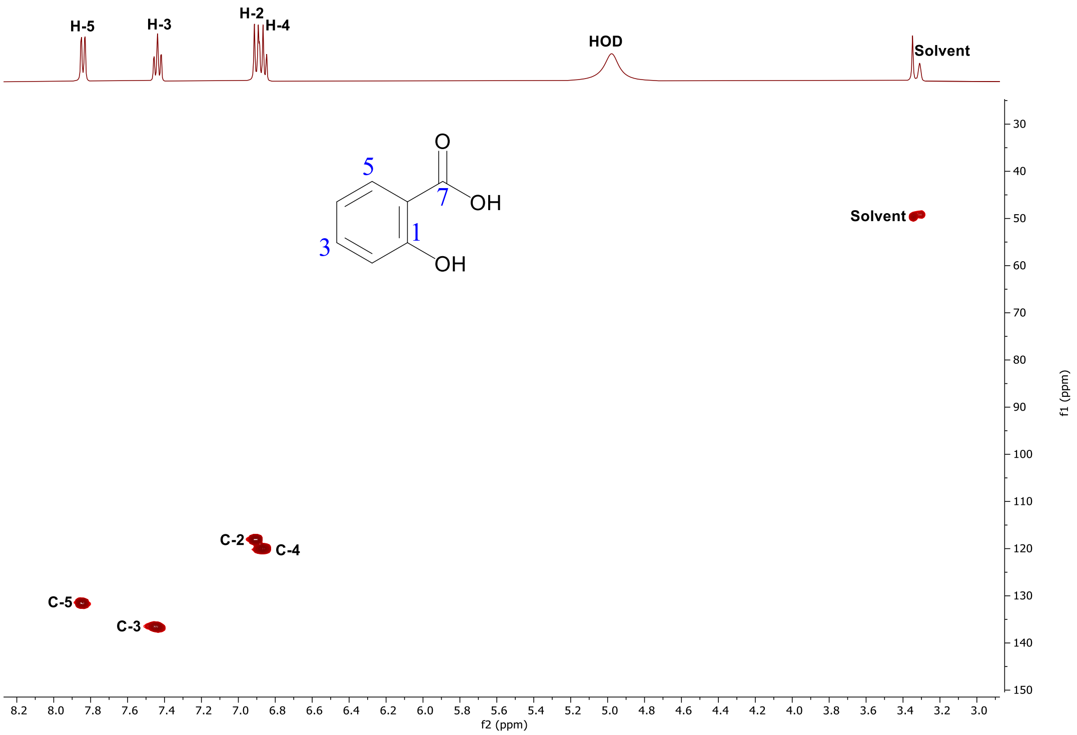

Figure S36. HSQC spectrum of **7.**

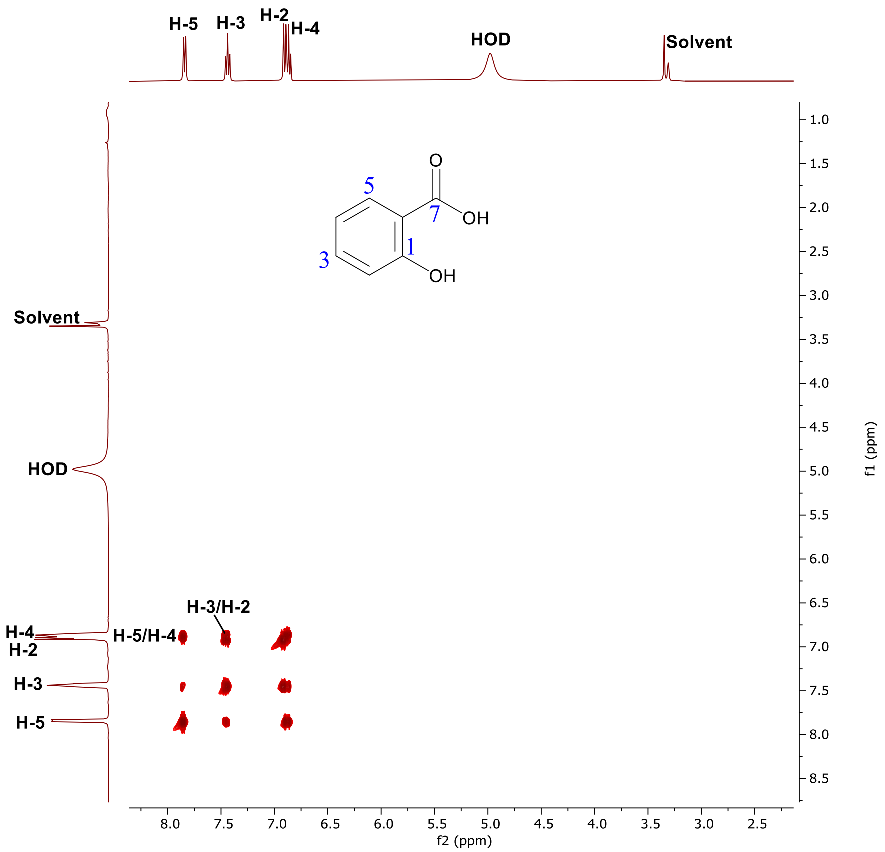

Figure S37. COSY spectrum of **7.**

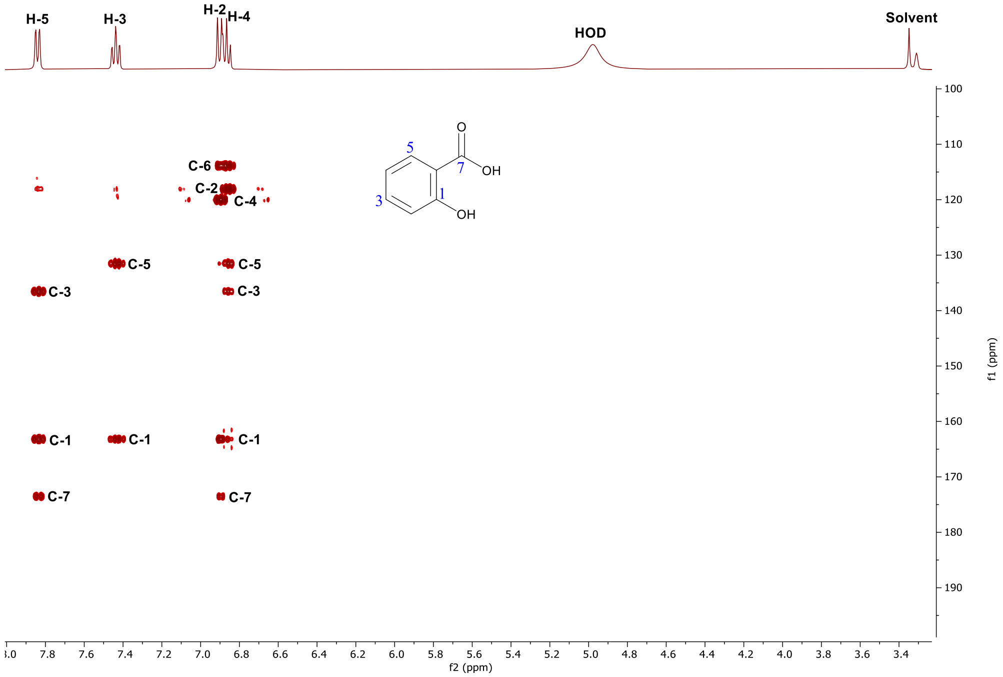

Figure S38. HMBC spectrum of **7**.

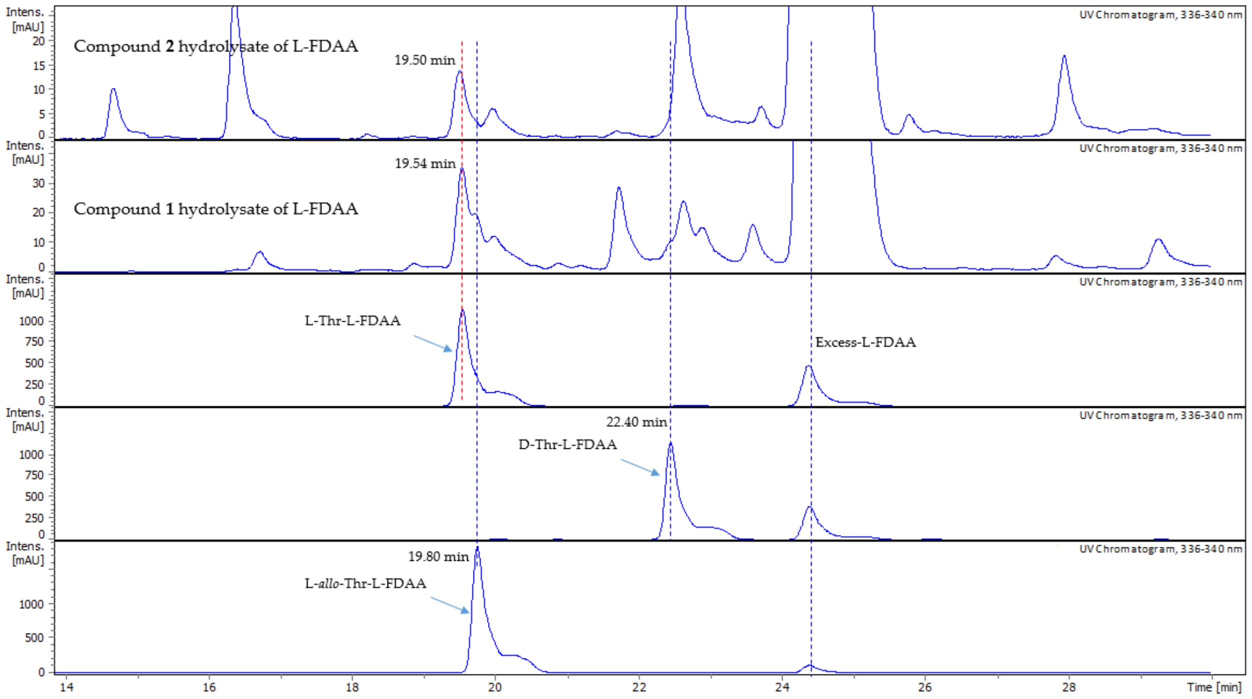

Figure S39. HPLC profiles of L-FDAA derivatised amino acid standards and L-FDAA derivatised hydrolysates of compounds **1** and **2**.

Experimental procedures

Marfey's analysis

The Marfey's analysis was carried out for compounds **1** and **2** in a manner analogous to the procedure mentioned in [1].

Dose-response cytotoxicity assays.

Specific details for each cell line are given below. In general: parasites were counted using CASY TT Cell Counter and mammalian cells were counted using a haemocytometer. Alamar blue cell viability assays were performed [2], whereby cells were incubated in the appropriate media containing the treatment compound (dissolved in DMSO) in serial dilution, after which the Alamar blue cell viability reporter was added, and the cells incubated further. Fluorescence was recorded using a FLx 800 plate reader (BioTek) with excitation wavelength 530–535 nm and emission wavelength at 590-610 nm using Gen5 Reader Control 2.0 Software (BioTek). EC_{50} values were determined using a 4-parameter non-linear logistic regression equation using GraFit 5.0 (Erithacus Software). SD values were calculated based on curve fitting to 3 or 4 biological replicates performed in parallel.

Bloodstream trypomastigote form *T. brucei* were maintained in HMI-11 medium as described above. Mid-log-phase cells were added to compound (2-fold serially diluted in HMI-11 growth medium) to a density of 1×10^3 cells/well (final volume 200 µL) and incubated at 37 ºC with 5% v/v CO_2 for 72 h. After which, 10 µL alamar blue (1.1 mg/mL resazurin sodium salt in PBS) was added to all wells and the cells incubated for a further 7 h before recording fluorescence [3].

Epimastigote *T. cruzi* CL Brener was maintained in RTH growth medium as described above. Mid-log-phase cells were added to compound (2-fold serially diluted in RTH growth medium) to a density of 5×10^5 cells/well and incubated at 28 ºC (final volume 200 µL) for 72 h. After which, 10 uL alamar blue (1.1 mg/mL resazurin sodium salt in PBS) was added to all wells and the cells incubated for a further 7 h before recording fluorescence [4].

Promastigote *L. major* strain Freidlin were maintained in M199 growth medium as described above. Mid-log-phase cells were added to compound (2-fold serially diluted in M199 growth medium) to a density of 2×10^4 cells/well (final volume 200 µL) and incubated at 28 ºC for 72 h. After which, 10 µL alamar blue (1.1 mg/mL resazurin sodium salt in PBS) was added to all wells and the cells incubated for a further 7 h before recording fluorescence [5].

Table S1. In-vitro activities of compounds **1–7**

**Compound ID**	**EC_{50} (µM)**	**EC_{50} (µM)**	**EC_{50} (µM)**
	Trypanosoma brucei	***Trypanosoma cruzi***	***Leishmania major***
**1**	>500	>500	>500
**2**	>500	>500	82 ± 5
**3**	>500	>500	>500
**4**	137	127	101
**5**	>500	>500	92 ± 4
**6**	>500	>500	>500
**7**	>500	>500	>500
Pentamidine	0.025 ± 0.003	-	-
Nifurtimox	-	2.78 ± 0.39	-
Miltefosine	-	-	5.28 ± 0.24

References

1. Oluwabusola, E. T.,; Adebisi, O. O.; Reyes, F.; Acquah, K. S.; De La Cruz, M.; Mweetwa, L. L.; Rajakulendran, J. E.; Warner, D. F.; Hai, D.; Ebel, R.; Jaspars, M. Beilstein J. Org. Chem. 2021, 17, 2390-2398.
2. Mikus, J.; Steverding, D. Parasitol. Int. 2000. 48, 265–269.
3. Cockram, P.E.; Dickie, E. A.; Barrett, M. P.; Smith, T. K. PLoS Negl. Trop. Dis. 2020, 14, e0008928.
4. Kanazawa, H.; Saavedra, O. M.; Maianti, J. P.; Young, S. A.; Izquierdo, L.; Smith, T. K.; Hanessian, S.; Kondo, J. ChemMedChem. 2018, 13, 1541-1548.
5. Garsi, J. B.; Hocine, S.; Hensienne, R.; Moitessier, M.; Denton, H.; Major, L. L.; Smith, T. K.; Hanessian, S. Bioorg. Med. Chem. Lett. 2024, 110, 129883.
